# Analysis of extracellular vesicle DNA at the single‐vesicle level by nano‐flow cytometry

**DOI:** 10.1002/jev2.12206

**Published:** 2022-04-04

**Authors:** Haisheng Liu, Ye Tian, Chengfeng Xue, Qian Niu, Chen Chen, Xiaomei Yan

**Affiliations:** ^1^ Department of Chemical Biology MOE Key Laboratory of Spectrochemical Analysis & Instrumentation Key Laboratory for Chemical Biology of Fujian Province Collaborative Innovation Center of Chemistry for Energy Materials College of Chemistry and Chemical Engineering Xiamen University Xiamen People's Republic of China

**Keywords:** DNA, DNase digestion, exosomes, extracellular vesicles, microvesicles, nano‐flow cytometry, single particle analysis

## Abstract

It has been demonstrated recently that extracellular vesicles (EVs) carry DNA; however, many fundamental features of DNA in EVs (EV‐DNA) remain elusive. In this study, a laboratory‐built nano‐flow cytometer (nFCM) that can detect single EVs as small as 40 nm in diameter and single DNA fragments of 200 bp upon SYTO 16 staining was used to study EV‐DNA at the single‐vesicle level. Through simultaneous side‐scatter and fluorescence (FL) detection of single particles and with the combination of enzymatic treatment, present study revealed that: (1) naked DNA or DNA associated with non‐vesicular entities is abundantly presented in EV samples prepared from cell culture medium by ultracentrifugation; (2) the quantity of EV‐DNA in individual EVs exhibits large heterogeneity and the population of DNA positive (DNA^+^) EVs varies from 30% to 80% depending on the cell type; (3) external EV‐DNA is mainly localized on relatively small size EVs (e.g. <100 nm for HCT‐15 cell line) and the secretion of external DNA^+^ EVs can be significantly reduced by exosome secretion pathway inhibition; (4) internal EV‐DNA is mainly packaged inside the lumen of relatively large EVs (e.g. 80–200 nm for HCT‐15 cell line); (5) double‐stranded DNA (dsDNA) is the predominant form of both the external and internal EV‐DNA; (6) histones (H3) are not found in EVs, and EV‐DNA is not associated with histone proteins and (7) genotoxic drug induces an enhanced release of DNA^+^ EVs, and the number of both external DNA^+^ EVs and internal DNA^+^ EVs as well as the DNA content in single EVs increase significantly. This study provides direct and conclusive experimental evidence for an in‐depth understanding of how DNA is associated with EVs.

## 1 INTRODUCTION

Extracellular vesicles (EVs) are nanoscale membrane vesicles secreted by almost all cell types to mediate intercellular communication via transferring proteins, nucleic acids and lipids from donor to recipient cells (Kalluri and LeBleu, 2020; Mathieu et al., 2019; Shah et al., 2018). Recent studies have demonstrated the presence of genomic DNA, mitochondrial DNA and even viral DNA in EVs (Cai et al., 2013; Fernando et al., 2017; Jabalee et al., 2018; Lazaro‐Ibanez et al., 2019; Malkin and Bratman, 2020; Saari et al., 2020; Sansone et al., 2017; Thakur et al., 2014). Through packaging and horizontal transfer of DNA, EVs play crucial roles in maintaining cellular homoeostasis, modulating immune responses and regulating tumour progression (Cai et al., 2016; Elzanowska et al., 2021; Kitai et al., 2017; Kurywchak et al., 2018; Lian et al., 2017; Sisquella et al., 2017; Takahashi et al., 2017; Torralba et al., 2018). Recently, liquid biopsy tests have been developed for tumour diagnosis based on DNA in EVs (EV‐DNA) (Allenson et al., 2017; Garcia‐Silva et al., 2021; Hur et al., 2018; Kahlert et al., 2014; Maire et al., 2021; Xu et al., 2018; Yang et al., 2017). Although the biological significance of EV‐DNA has been recognized, EV‐DNA has been less explored and many fundamental features remain controversial, such as whether DNA is associated with all or some EV subsets? Is EV‐DNA located in the lumen and/or on the surface of EVs? Is there any relationship between DNA content and EV size? Is EV‐DNA single‐stranded DNA (ssDNA) or double‐stranded DNA (dsDNA)?

Studies on EV‐DNA are normally conducted by extracting DNA from EV isolates, followed by abundance, fragment length and sequence assessment (Fernando, et al., 2017; Fischer et al., 2016; Kawamura et al., 2017; Lazaro‐Ibanez, et al., 2019; Nemeth et al., 2017; Saari, et al., 2020; San Lucas et al., 2016; Sansone, et al., 2017; Thakur, et al., 2014; Torralba, et al., 2018; Vagner et al., 2018; Zhang et al., 2018). By combining DNase enzymatic digestion with a Fragment Analyzer system, the relative abundance and localization (inside the lumen or associated with the surface of EVs) of DNA were investigated (Thakur et al., 2014). To clarify the heterogeneity of EV‐DNA between EV subpopulations, DNA analyses of EVs isolated by density gradient centrifugation or asymmetric flow field‐flow fractionation have been carried out (Jeppesen et al., 2019; Lazaro‐Ibanez et al., 2019; Zhang, et al., 2018). By using a bottom‐up iodixanol density flotation gradient, Lazaro–Ibanez et al. discovered that both high‐ and low‐density small EVs (sEVs) isolated from cell culture media contained DNA cargo, and high‐density sEVs carried more DNA than low‐density sEVs (Lazaro‐Ibanez et al., 2019). However, using a similar approach, Jeppesen et al. found that DNA in EV isolates was associated with non‐vesicular entities rather than EVs (Jeppesen et al., 2019). Although bulk analysis enables the identification of DNA in different EV subsets, the results can be controversial due to the fact that EV‐DNA cannot be differentiated from cell‐free DNA, including naked DNA or DNA associated with non‐vesicular particles co‐isolated with EVs (Jeppesen et al., 2019; Tian et al., 2020; Zhang et al., 2019). Since EVs vary largely in size and cargo content, single‐particle techniques are urgently needed to decipher the large intrinsic heterogeneity of EV‐DNA and distinguish EV‐DNA from cell‐free DNA or other contaminants (Choi et al., 2019; Jeppesen et al., 2019). However, the nanoscale particle size of EVs (with a majority population sized <100 nm) and the low content of EV‐DNA render it a great challenge.

Over the past decade, our laboratory has been working on the development of a highly sensitive nano‐flow cytometer (nFCM). It has achieved light‐scattering detection of single EVs, viruses, silica nanoparticles and gold nanoparticles as small as 40, 27, 24 and 7 nm, respectively (Lian et al., 2019; Ma et al., 2016; Tian et al., 2020; Tian et al., 2018; Zhu et al., 2014). For fluorescence (FL) detection, single R‐phycoerythrin molecules were detected with a signal‐to‐noise ratio of 17, and the limit of detection for organic dyes was determined to be three Alexa Fluor 532 molecules (Yang et al., 2009; Zhu et al., 2014). In the present study, we attempted to analyse both the external and internal EV‐DNA at the single‐vesicle level by combining enzymatic digestion with nFCM. The percentage of DNA^+^ EVs along with the DNA content distribution versus EV size, the differentiation between ssDNA and dsDNA, the association of EV‐DNA and histone proteins and the alteration of DNA content upon anticancer drug treatment were investigated.

## 2 MATERIALS AND METHODS

### 2.1 Cell culture

The human colorectal cancer cell line (HCT‐15) and a normal human colon fibroblast cell line (CCD‐18Co) were purchased from the American Type Culture Collection (ATCC). The human nasopharyngeal epithelial cell line (NP69) and nasopharyngeal carcinoma cell line (C666‐1) were generously provided by Professor George Tsao (University of Hong Kong, China). HCT‐15 and C666‐1 cells were cultured in RPMI‐1640 medium (Gibco, 11875‐093), and CCD‐18Co cells were cultured in minimal essential medium (MEM) (Gibco, 41500‐067). All media were supplemented with 10% FBS and penicillin‐streptomycin (Invitrogen). The FBS used above was depleted of EVs by ultracentrifugation at 100,000 × *g* for 18 h at 4°C (Beckman Coulter X‐90 centrifuge, SW41 Ti rotor). NP69 cells were cultured in serum‐free keratinocyte medium supplemented with human recombinant epidermal growth factor (0.1–0.2 ng/ml) (TBD Science, Tianjing, China). Cells were grown in EV‐depleted medium until they reached a confluence of approximately 90% (after approximately 24 h) before the culture medium was collected. To investigate the effect of anti‐tumour drugs on EV‐DNA, HCT‐15 cells were treated with 100‐μM etoposide (MedChemExpress, HY‐13629), 20‐μM topotecan (MedChemExpress, HY‐13768A) or 10‐μM SN‐38 (MedChemExpress, HY‐13704) in six‐well tissue culture plates for 48 h before the culture medium was collected. The culture medium was centrifuged at 800 × *g* for 5 min at 4°C to pellet the cells. The supernatant was centrifuged at 2000 × *g* for 10 min at 4°C to remove cellular debris and is called the conditioned cell culture medium (CCCM) in the present study.

### 2.2 EV isolation for nFCM analysis

Freshly prepared CCCMs of HCT‐15 cells (1 ml) were centrifuged at 100,000 × *g* for 17 min at 4°C (Beckman Coulter Optima Max‐XP ultracentrifuge, TLA 120.2 rotor). The pellet was washed with 1 ml of PBS, followed by a second ultracentrifugation at 100,000 × *g* for 17 min at 4°C. Afterwards, the supernatant was discarded, and the EVs were resuspended in 100 μl of PBS. All PBS used in this study was filtered through a 220‐nm filter. Noting that centrifuging at 100,000 ×  *g* for 17 min with Beckman Coulter Optima Max‐XP ultracentrifuge equipped with a TLA 120.2 rotor offers the same fractionation effect as that of centrifuging at 100,000 ×  *g* for 120 min with Beckman Coulter XE‐90K Ultracentrifuge equipped with an SW41 Ti rotor (https://www.mybeckman.uk/centrifuges/rotors/calculator). For the purity assessment experiment, 10 μl of 10% Triton X‐100 (Sigma‐Aldrich, X100) was added to 90 μl of EV preparation with a particle concentration of approximately 6 × 10^9^ particles/ml. After 30 min of incubation at 4°C, the treated sample was diluted 20‐fold prior to the nFCM analysis. Note that concentration of EV preparations was measurement by nFCM by employing 100‐nm Yellow‐Green FluoSpheres with known particle concentration (Thermo Fisher, F8803) as an external standard via the equation *C*_EVs_ = (*N*_EVs_/*N_f_*) × *C_f_*, where *C*_EVs_, *C_f_*, *N*_EVs_, and *N_f_* represent EVs concentration, FluoSphere concentration, the event number of EVs counted in 1 min, and the event number of FluoSphere counted in 1 min, respectively.

### 2.3 DNA fragments and EV‐DNA staining for nFCM analysis

A mixture of three different lengths of dsDNA fragments containing 0.1 ng/ml of 400‐bp fragments (Thermo Fisher, SM1631), 0.4 ng/ml of 2000‐bp fragments (Thermo Fisher, SM1701) and 1.2 ng/ml of 5000‐bp fragments (Thermo Fisher, SM1731) was stained with 6‐μM SYTO 16 for 20 min at 37°C before nFCM analysis. A 100‐μl aliquot of the EV preparation with a particle concentration of approximately 3 × 10^8^ particles/ml was treated with 0.2‐U/μl RNase‐free DNase I (Takara, 2270A), 2‐U/μl S1 nuclease (ThermoFisher, EN0321), 1 × dsDNase (ThermoFisher, EN0771) or 200‐μg/ml proteinase K (ThermoFisher, AM2548) for 30 min at 37°C. SYTO 16 (ThermoFisher, S7578) in PBS was added to obtain a final dye concentration of 6 μM. The EV sample was incubated for 20 min at 37°C prior to the nFCM analysis.

### 2.4 EdU staining for nFCM analysis

A Click‐iT™ Plus EdU Alexa Fluor™ 488 Flow Cytometry Assay Kit (Thermo Fisher, C10632) was used in the present study. The reagents were prepared according to the manufacturer's instructions. First, 0.6 μl of 10‐mM EdU in DMSO or 0.6‐μl DMSO (negative control) was added to 3‐ml cell culture medium. The HCT‐15 cells were cultured with these media at 37°C for 72 h. Then, 3 ml of culture medium from 0.02% DMSO‐ or EdU‐treated cells was collected and centrifuged at 800 × *g* for 5 min at 4°C, 2000 × *g* for 10 min at 4°C and 100,000 × *g* for 17 min at 4°C. The EV pellet was resuspended in 100 μl of 4% paraformaldehyde (PFA) in PBS (component D) and incubated at room temperature for 30 min. The PFA was washed away with 1‐ml PBS by ultracentrifugation at 100,000 × *g* for 17 min at 4°C. Subsequently, the EV pellet was resuspended in 100 μl of 1× saponin‐based permeabilization and wash reagent (component E) and incubated for 15 min at room temperature. Finally, 500‐μl click‐iT reaction cocktail with Alexa Fluor™ 488 picolyl azide (component B), copper protectant (component F) and buffer additive mixed in PBS was added to the EV sample. The mixture was incubated for 30 min at room temperature and protected from light. After two washes by ultracentrifugation at 100,000 × *g* for 17 min at 4°C with 1 ml of 1× saponin‐based permeabilization and wash reagent, the sample was resuspended in 100‐μL PBS for nFCM analysis.

### 2.5 EV‐DNA extraction for fragment length analysis by Agilent 4200 TapeStation System

Freshly prepared CCCM from HCT‐15 cells (60 ml) was divided into equal portions in five tubes and centrifuged at 100,000 × *g* for 2 h at 4°C (Optima XE‐90 ultracentrifuge with a SW 41Ti rotor, Beckman Coulter). All EV pellets were combined into a centrifuge tube and suspended in 400 μl of PBS. The EV suspension was divided equally into two samples. One sample was treated with 0.2‐U/μl RNase‐free DNase I (Takara, 2270A) at 37°C for 30 min to digest cell‐free DNA and external DNA associated with EVs. Then, the EV samples with or without DNase I treatment were washed once by centrifugation at 100,000 × *g* for 2 h at 4°C. The EV pellet was resuspended in 200‐μl PBS, and EV‐DNA was extracted using the QIAamp DNA Mini kit (QIAGEN, 51304) and eluted with 50 μl of PBS. DNA quantity was measured on a Qubit 4.0 fluorometer (Thermo Fisher) using the Qubit™ 1× dsDNA HS Assay Kit (Thermo Fisher, 1992693). The DNA fragment length distribution was assessed by using the Agilent 4200 TapeStation System (Agilent) with the genomic DNA ScreenTape Assay and reagents for sizes ranging from 200 bp to >60,000 bp (Agilent, 5067‐5365).

### 2.6 DNase digestion of EVs upon fixation and permeabilization for nFCM analysis

Twenty millilitres of freshly prepared CCCM of HCT‐15 cells were divided equally into two tubes and centrifuged at 100,000 × *g* for 2 h at 4°C in a Beckman Coulter XE‐90K Ultracentrifuge using an SW 41 Ti rotor. The pellets were combined into one tube, resuspended in 100‐μl PBS containing 0.2‐U/μl RNase‐free DNase I, and incubated for 30 min at 37°C to eradicate cell‐free DNA and external DNA associated with the outer membrane of EVs. DNase I was washed away with 12‐ml PBS via ultracentrifugation at 100,000 × *g* for 2 h at 4°C. For EV fixation, 100 μl of 4% PFA (Alfa Aesar, W13C179) was used to resuspend the EVs, and the mixture was incubated for 30 min at room temperature. The PFA was washed away with 1‐ml PBS by ultracentrifugation at 100,000 × *g* for 17 min at 4°C (Beckman Coulter Optima Max‐XP ultracentrifuge, TLA 120.2 rotor). Subsequently, 50 μl of 0.5% Triton X‐100 in PBS was added and incubated for 2 min at room temperature to permeabilize the EV membrane (Song et al., 2014). The EV sample was immediately diluted 20‐fold with 950‐μl PBS to prevent potential damage to the EV structure with the 0.5% of Triton X‐100. Triton X‐100 was washed by ultracentrifugation at 100,000 × *g* for 17 min at 4°C, and the EV pellet was resuspended in 80 μl of PBS. The EV suspension was divided equally into four samples. Then, these samples were treated without or with 2‐U/μl RNase‐free DNase I, 5× dsDNase or S1 nuclease at 37°C for 4 h. Finally, each sample was diluted to 50 μl in PBS and incubated with 6‐μM SYTO 16 for 20 min at 37°C before nFCM analysis.

### 2.7 Immunofluorescent staining for nFCM analysis

PE‐conjugated mouse anti‐human CD9 antibody (clone M‐L13), PE‐conjugated mouse anti‐human CD63 antibody (clone H5C6), PE‐conjugated mouse anti‐human CD81 antibody (clone JS‐81) and PE‐conjugated mouse IgG1, κ (clone MOCP‐21) were purchased from BD Biosciences. For immunofluorescent staining of EV transmembrane proteins, EVs were isolated from 1‐ml CCCM of HCT‐15 cells by centrifugation at 100,000 × *g* for 17 min at 4°C (Beckman Coulter Optima Max‐XP ultracentrifuge, TLA 120.2 rotor), and the pellet was resuspended in 50‐μl PBS. To a 50‐μl EV sample with a particle concentration of approximately 6 × 10^8^ particles/ml, 20 μl of PE‐conjugated antibody against CD9, CD63, CD81 or IgG1 was added. The mixture was incubated at 37°C for 30 min, and then unbound antibodies were washed twice with 1‐ml PBS by ultracentrifugation at 100,000 × *g* for 17 min at 4°C. The pellet was resuspended in 50‐μl PBS for nFCM analysis.

Regarding immunofluorescent staining of EV luminal proteins, mouse anti‐human β‐actin antibody (Proteintech, 60008‐1‐1g) and mouse anti‐human histone H3 antibody (Thermo Fisher, MA531759) were labelled with Alexa Fluor 488 (AF488) following the instruction of AF488 Microscale Protein Labeling Kit (Molecular Probes, A30006). EVs were isolated from 5‐ml CCCM of HCT‐15 cells by centrifugation at 100,000 × *g* for 17 min at 4°C (Beckman Coulter Optima Max‐XP ultracentrifuge, TLA 120.2 rotor), and the pellet was resuspended in 100 μl 4% PFA and incubated at room temperature for 30 min. After fixation, the PFA was washed off with 1‐ml PBS by ultracentrifugation at 100,000 × *g* for 17 min at 4°C. Subsequently, the EV pellet was resuspended in 100 μl of 0.2% Tween 20 (Sigma‐Aldrich, P9416) and incubated for 30 min at room temperature. After permeabilization, Tween 20 was washed away with 1‐ml PBS by ultracentrifugation at 100,000 × *g* for 17 min at 4°C. Then, the EV pellet was resuspended in 50‐μL PBS, and the particle concentration was approximately 6 × 10^8^ particles/ml. Then, 5 μg/ml of AF488‐conjugated mouse anti‐human β‐actin antibody or AF488‐conjugated mouse anti‐human histone H3 antibody was added. The mixture was incubated at 37°C for 30 min and then washed twice with 1‐ml PBS by ultracentrifugation at 100,000 × *g* for 17 min at 4°C (Beckman Coulter MAX‐XP centrifuge, TLA‐120.2 rotor). The pellet was resuspended in 50‐μl PBS for nFCM analysis.

### 2.8 Western blotting

The protein concentration of EV preparations was measured using a Pierce ™ BCA protein assay kit (Thermo Fisher, 23227). For each sample, the protein concentration was adjusted to 10 μg/20 μl, and 10 μg of protein was loaded onto a 15% polyacrylamide gel. Following electrophoresis, the proteins were transferred from the gel onto a polyvinylidene fluoride membrane (PVDF, Millipore) using a Trans‐Blot Turbo Transfer System (Bio‐Rad). The membrane was blocked with 5% non‐fat dry milk in TBST for 30 min at room temperature and incubated with primary rabbit anti‐human CD9 antibody (Abcam, ab92726), rabbit anti‐human syntenin antibody (Abcam, ab133267), rabbit anti‐human calnexin antibody (Abcam, ab213243), rabbit anti‐human beta‐tubulin antibody (Abcam, ab179513) or primary rabbit anti‐human histone H3 antibody (Cell Signaling Technology, 4499T) overnight at 4°C. Following incubation with HRP‐conjugated goat anti‐rabbit immunoglobulin G (IgG) (1:3000, Abcam, ab6721), the blot was developed using chemiluminescent reagents from Advansta. Images were captured using an Amersham Imager 600 (GE Healthcare Life Sciences).

### 2.9 Immuno‐gold labelling of histone H3 in EVs and transmission electron microscopy (TEM)

A 20‐μL aliquot of the EV preparation with a concentration of approximately 10^11^ particles/ml was mixed with 20 μl of 4% PFA and incubated at room temperature for 15 min. Then, the fixed EV preparation was divided evenly into two samples, and each sample was placed on a formvar/carbon‐coated grid and allowed to settle for 5 min. For immuno‐gold staining, both grids were placed in a blocking buffer (PBS containing 1% BSA) for 30 min. Without rinsing, grid A was immediately placed in 20 μl of 5 μg/ml rabbit antihuman histone H3 antibody and incubated at room temperature for 1 h. Meanwhile, grid B was exposed to 20 μl of 5 μg/ml rabbit IgG antibody (abcam, ab172730) to serve as a control. Both grids were rinsed with PBS and placed in 20 μl of 5 μg/ml goat antirabbit IgG (H+L) (Boster, GA1013) conjugated to 10 nm colloidal gold for 30 min at room temperature. The grids were rinsed with PBS and placed in 1% glutaraldehyde for 2 min at room temperature. After rinsing with PBS and distilled water, the grids were allowed to dry. Both samples were negative‐stained with 2% phosphotungstic acid for 1 min at room temperature. Grids were imaged using a Tecnai G2 Spirit BioTwin TEM operating at 120 kV.

### 2.10 nFCM analysis

The laboratory‐built nFCM reported previously was used in the present study (Tian et al., 2020; Tian et al., 2018) except that the excitation laser was replaced with a 20‐mW 488‐nm laser (OBIS 488LS, Coherent). Briefly, the light emitted by individual nanoparticles or EVs was collected perpendicularly to both the laser beam and the sample stream by an infinity‐corrected microscope objective (Olympus ULWD MSPlan 50×, 0.55 N.A.). Two single‐photon counting avalanche photodiodes (APDs) were used for the simultaneous detection of side scatter (bandpass filter: FF01‐488/6) and green FL (bandpass filter: FF01‐525/45) of individual particles/EVs, respectively. The output signals from the APD detectors were counted using a National Instruments DAQ card (PCIe‐6321). A custom programme written with LabVIEW 2012 software was used for data acquisition and processing, and the bin width was set to 100 μs. The threshold levels for both the peak height (a digital discriminator level set to 3 times the standard deviation of the background) and the peak width of 0.2 and 0.3 ms were set as the criteria for burst (or peak) identification of SSC and FL signal, respectively. For each burst that satisfied the criteria, the integrated number of photons (background subtracted) was stored as the burst area for the histogram or dot‐plot construction. Unless stated otherwise, each distribution histogram or dot‐plot was derived from the data collected over 1 min. For nFCM analysis, the sample stream is completely illuminated within the central region of the focused laser beam, and the detection efficiency was approximately 100%, which led to accurate particle concentration measurements via single‐particle enumeration (Yang et al., 2009; Zhu et al., 2010). The detection volume of the nFCM used in the present study was approximately 25 fL. Based on Poisson statistics, when the concentration of particles is ∼1 × 10^9^/ml, the probability that two particles will coincide with the probe volume is 0.7%. Ultrapure water supplied by an ultrapure water system (PURELAB Ultra FLC00006307, ELGA) served as the sheath fluid via the gravity feed.

## 3 RESULTS

### 3.1 Isolation and characterization of EVs

**FIGURE 1:**
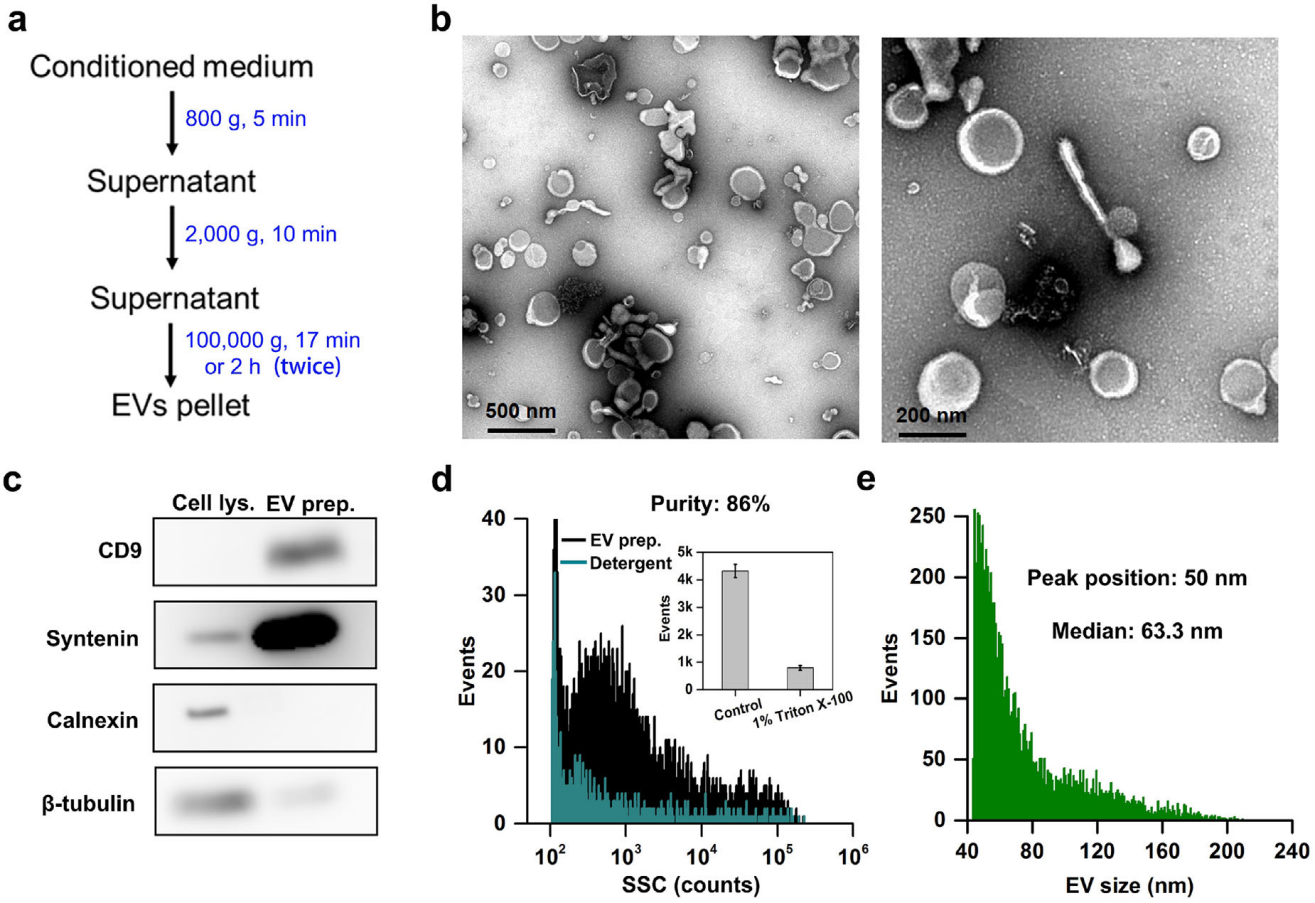
Isolation and characterization of extracellular vesicles (Evs). (a) Procedure for EV isolation from the conditioned cell culture medium of human colorectal cancer HCT‐15 cells by ultracentrifugation. (b) Representative transmission electron microscopy (TEM) micrographs of an EV isolate, the scale bars are 500  and 200 nm, respectively, for (i) and (ii). (c) Immunoblots of a cell lysate and an EV preparation. (d) Side‐scatter (SSC) distribution histograms for an EV preparation before (black line) and after Triton X‐100 treatment (cyan line), along with the bar graph of events rate detected in 1 min for both samples (inset). The error bar represents the standard deviation (s.d.) of three replicate experiments. (e) Histogram of particle size with a bin width of 1 nm for an EV sample isolated from CCCM of HCT‐15 cells by ultracentrifugation

Supernatants collected from cultured HCT‐15 cells were used as the model system, and EVs were isolated from CCCM by 100,000 × *g* ultracentrifugation (Figure 1(a)). The morphology of EVs and the presence of proteins in EV preparation were assessed by TEM and Western blotting, respectively. Figure 1(b, c) demonstrates a good recovery of EVs. In order to quantitatively characterize the purity of EV preparation, the laboratory‐built nFCM that can detect side‐scattered light of single EVs as small as 40 nm was used to enumerate and compare the event rates detected in 1 min before and after Triton X‐100 treatment (Tian et al., 2020; Tian et al., 2018). Figure 1(d) shows the side‐scatter (SSC) burst area distribution histogram of a typical EV preparation before and after detergent treatment. The event rate of PBS measured at the same settings was deducted from each EV sample, and the purity of the EV isolate was measured to be 86%. To measure the particle size of EV preparation, a mixture of monodisperse silica nanoparticles (SiNPs) of five different diameters ranging from 47 to 123 nm (47, 59, 74, 95 and 123 nm) was analysed using nFCM at the same instrument settings as those used for EV analysis. The SSC distribution histogram is shown in Figure S1(a). Considering the refractive index difference between the SiNPs (1.463) and EVs (1.400) at 488‐nm excitation, the intensity ratio between light scattered by a SiNP to that of an EV of the same particle size was calculated based on the Mie theory for every size of the SiNP standard. These ratios were used as the correction factors to derive the calibration curve between the scatted light intensity and particle size of EVs from the data of SiNPs (Tian et al., 2020; Tian et al., 2018). The EV size distribution histogram shown in Figure 1(e) indicates that EVs isolated from the CCCM of HCT‐15 cells mainly fell in the size range of 40–200 nm. The peak position and median values were measured to be 50  and 63.3 nm, respectively. These values agreed well with our previously reported values of 50  and 64.5 nm for EVs isolated from the CCCM of HCT‐15 cells using ultracentrifugation (Tian et al., 2018).

### 3.2 Cell‐free DNA exists abundantly in EV samples prepared from cell culture medium by ultracentrifugation

**FIGURE 2:**
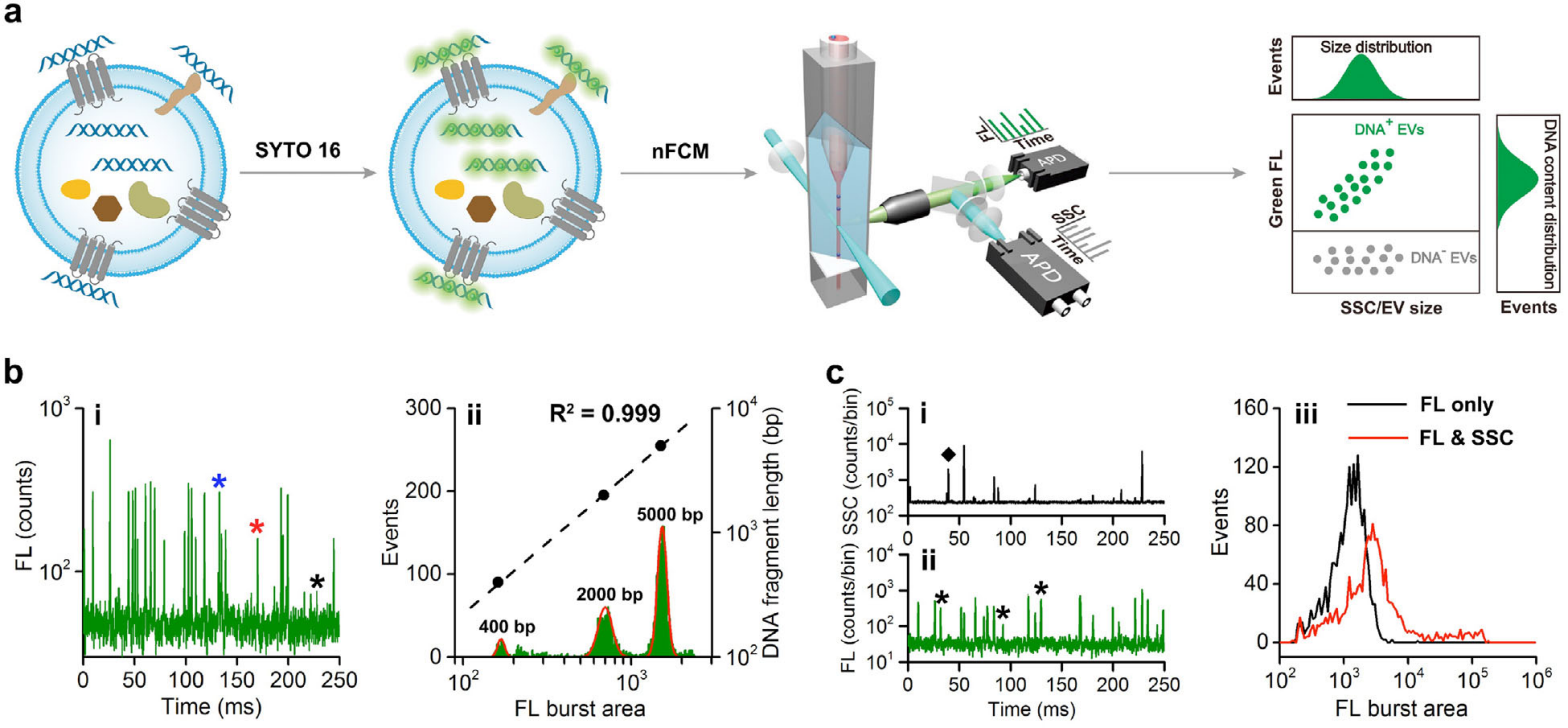
Principle and sensitivity for EV‐DNA analysis at the single‐extracellular vesicles (EV) level by nano‐flow cytometer (nFCM). (a) Schematic depiction of EV‐DNA staining and nFCM analysis. (b) nFCM analysis of a double‐stranded DNA (dsDNA) mixture with three different fragment lengths upon SYTO 16 staining. (i) Typical fluorescence (FL) burst traces in a 250‐ms segment with *, *, and * denoting 400‐bp, 2000‐bp and 5000‐bp fragments, respectively. (ii) FL burst area distribution histogram and the correlation of the centroids of the burst areas obtained from the fitted Gaussian curve (red line) with known DNA fragment lengths. (c) Representative SSC (i) and FL (ii) burst traces and the FL burst area distribution histograms of events exhibiting FL only signal and events exhibiting concurrent FL and SSC signals (iii) for an EV preparation derived from the CCCM of HCT‐15 cells by ultracentrifugation and stained with SYTO 16

As schematically shown in Figure 2(a), isolated EVs were labelled with a membrane‐permeable nucleic acid stain, SYTO 16 and analysed on the nFCM. Both the SSC and green FL signals emitted from individual EVs were detected simultaneously. By analysing thousands of individual EVs in 1–2 min, a bivariate dot‐plot of nucleic acid green FL intensity versus SSC intensity can be quickly obtained. To enable quantitative detection of EV‐DNA with high sensitivity and selectivity, six nucleic acid stains were screened, including SYTO 9, SYTO 13, SYTO 16, PicoGreen, SYBR Green I and SYTO 82. The emission spectra of these dyes bound with DNA or RNA were measured on a spectrofluorometer, and these data indicated that SYTO 16 provided the most selective binding to DNA along with good FL enhancement (Figure S2).

To examine the sensitivity of nFCM for DNA detection, a mixture containing 400, 2000 and 5000‐base pairs (bp) of DNA fragments was stained with SYTO 16 and analysed by nFCM at the level of single DNA fragment. A representative FL burst trace is shown in Figure 2(b) (i), which reveals that the peak heights clustered around three different amplitude levels, which corresponded with the three differently sized DNA fragments. The FL burst area distribution histogram displayed in Figure 2(b) (ii) indicates that single DNA fragments of 400 bp in length can be well resolved from the background, and a linear correlation (*R* ^2^ = 0.999) between the fragment length and FL intensity was obtained. The detection limit was calculated to be 185 bp based on three times the standard deviation of the background and a peak width of three bins (0.3 ms). These data not only confirmed the stoichiometric DNA staining by intercalating dye molecule (Yan et al., 1999; Yan et al., 2000), but also indicated the great sensitivity of nFCM for the quantitative analysis of DNA content in single EVs. Note that DNA of a single EV is constituted by DNA fragments of different lengths and each length of the DNA fragment could have different copy numbers. Once the total DNA fragment length in a single EV is larger than ∼200 bp, EV‐DNA can be clearly distinguished from the background by nFCM.

We then analysed EV isolates stained with SYTO 16 by nFCM at the single‐vesicle level according to the MIFlowCyt‐EV guideline (Welsh et al., 2020). Figure S3 shows the representative SSC and FL burst traces of assay controls (buffer only, buffer with reagent and unstained EV samples) analysed at the same instrument settings as stained EV samples. Figure 2(c) shows representative SSC and FL burst traces of a stained EV sample. Compared with events with concomitant FL and SSC signals, there were more events (with several of them denoted with asterisk *) showing only FL signal. These events can be naked DNA or DNA associated with non‐vesicular entities such as protein assemblies co‐isolated with EVs of which the SSC signal is undetectable by the nFCM. These DNA are denoted as cell‐free DNA thereafter. Meanwhile, events with only SSC signal but no concurrent FL signal (denoted by ^♦^) were observed occasionally as shown in Figure 2(c). These events can be attributed to DNA‐negative (DNA^–^) EVs or impurity particles in the buffer or sheath fluid. Using PBS filtered through a 220‐nm membrane as the sample, the event rate for impurity particles was measured as 217 ± 7/min. The FL burst area distribution histograms for events with FL only or with concurrent FL and SSC signals (FL and SSC) are displayed in Figure 2(c) (iii). The median FL intensities of these two populations were 1280 and 2707, respectively. In three replicate experiments, the population ratio of the events with FL only among all the events with detectable FL signal was 61.6 ± 2.5%, which was even higher than for concurrent FL and SSC signals (38.8 ± 2.5%). An additional nFCM analysis of lambda DNA fragments (48.5 kbp) before and after 100,000 × *g* ultracentrifugation indicated that naked DNA fragments could be well retained in the solution phase upon ultracentrifugation (Figure S4).

### 3.3 There exist two populations of EVs with respect to EV‐DNA localization and EV size

**FIGURE 3:**
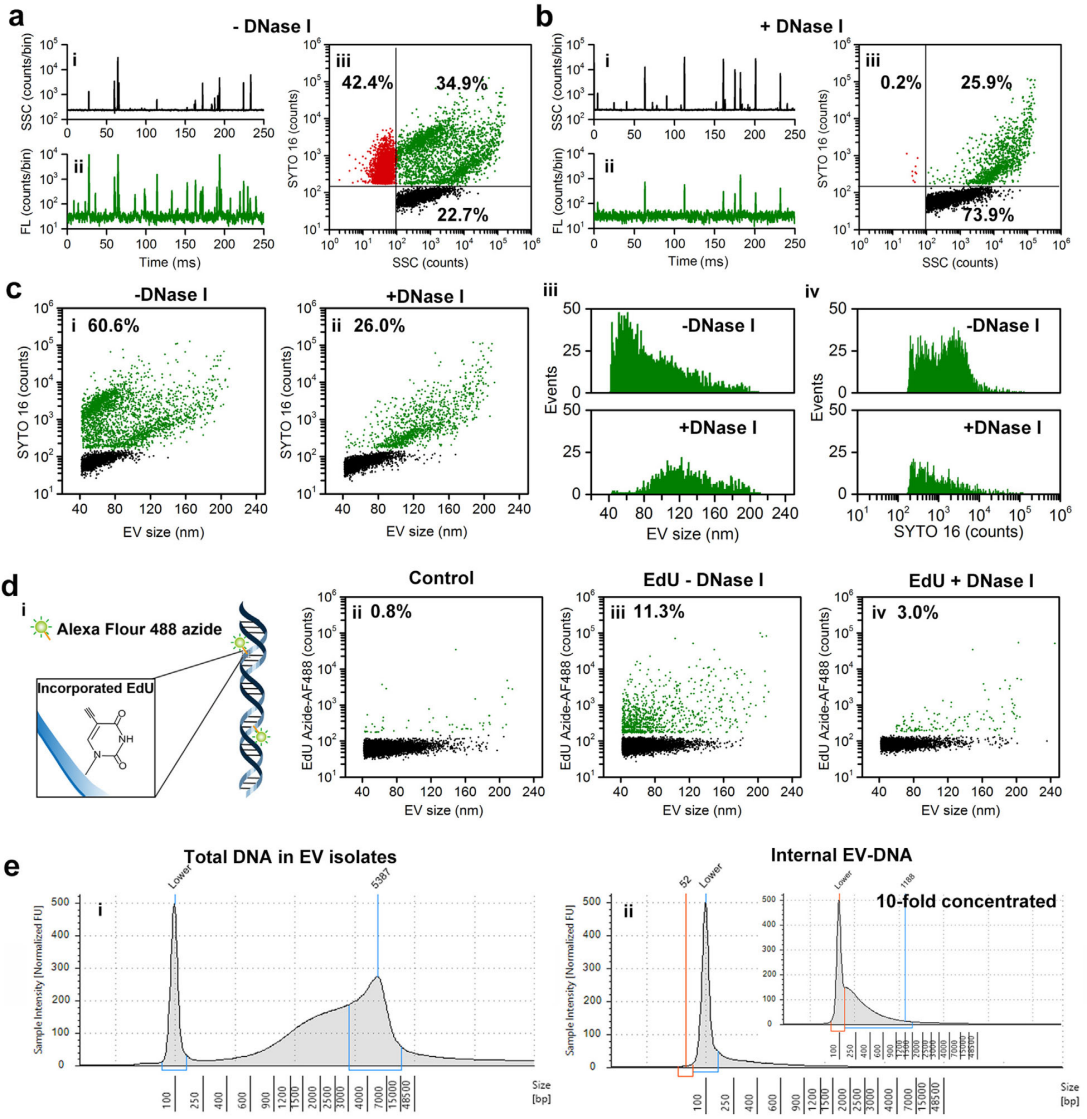
Characterization of DNA in EVs (EV‐DNA) by combining nano‐flow cytometer (nFCM) and DNase I digestion. (a, b) nFCM analysis of SYTO 16‐stained extracellular vesicles (EV) preparation from the conditioned cell culture medium (CCCM) of HCT‐15 cells by ultracentrifugation before (a) and after (b) DNase I digestion. (i, ii) Representative side‐scatter (SSC) (i) and FL (ii) burst traces; and (iii) bivariate dot‐plot of SYTO16 fluorescence versus SSC. (c) Bivariate dot‐plots of SYTO 16 fluorescence versus EV size for events with detectable SSC signal of the EV sample shown in (a) before (i) and after (ii) DNase I treatment along with the distribution histograms of EV size (iii) and SYTO 16 fluorescence (iv). (d) nFCM analysis of AF488 azide‐stained EV preparations by ultracentrifugation from CCCM of HCT‐15 cells cultured in the absence or presence of EdU. (i–iv) The labelling principle of incorporated EdU in EV‐DNA by AF488 azide via click chemistry (i), and the bivariate dot‐plots of EdU‐azide‐AF488 fluorescence versus EV size for EVs isolated from the CCCM of HCT‐15 cells cultured with 0.02% DMSO (reagent control) (ii), 20‐μM EdU (iii), or 20‐μM EdU and upon DNase I treatment (iv). (e) Chip‐based capillary electrophoresis of EV‐DNA by Aglient 4200 TapeStation system using Genomic DNA ScreenTape assay without (i) and with (ii) DNase I treatment

Figure 3(a) shows the representative SSC and FL burst traces and the bivariate dot‐plot of SYTO 16 FL intensity versus SSC for an EV isolate. Similar to Figure 2(c) (i) and (ii), three distinct populations can be identified, especially in the statistically significant dot‐plot of Figure 3(a) (iii). The events of FL only, SSC only and FL with concurrent SSC represent cell‐free DNA, debris or DNA^–^ EVs and DNA^+^ EVs, respectively. For DNA^+^ EVs, an approximately three orders of magnitude variation in DNA content was observed for individual EVs. Moreover, the DNA^+^ EVs can be separated along the diagonal into two sections, namely SSC‐low/SYTO 16‐high and SSC‐high/SYTO 16‐low. According to the MIFlowCyt‐EV guideline, detergent control can be used to determine whether detected events are EVs or non‐EV entities (Welsh et al., 2020). Figure S5 shows that after using 1% triton X‐100 to lyse EVs in the EV preparation, the event rate of DNA^+^ particles (with detectable SSC signal) dropped from 2907 ± 184 to 286 ± 138, and the event rate of DNA^–^ particles dropped from 2231 ± 154 to 307 ± 134. Noting that this Triton X‐100 treatment basically will not denature the vesicle‐free DNA or breaks down DNA aggregation (Figure S6). These results suggest that 90% of the DNA^+^ events detected by nFCM were EVs rather than DNA associated large non‐vesicular entities. Meanwhile, 86% of the DNA^–^ events were DNA^–^ EVs instead of impurity particles.

To confirm the presence of DNA attached to the outer membrane of EVs, DNase I, an endonuclease that non‐specifically digests both ssDNA and dsDNA by hydrolysing phosphodiester bonds, was used to treat the EV preparation before SYTO 16 staining and nFCM analysis. Through optimization, 0.2‐U/μl RNase‐free DNase I was used to treat the EV preparation (Figure S7). The representative SSC and FL burst traces (Figure 3(b) (i) and (ii)) indicate that upon DNase I treatment, the originally abundant FL only events vanished as compared to Figure 3(a) (i) and (ii). The statistically significant bivariate dot‐plot of SYTO 16 FL intensity versus SSC (Figure 3(b) (iii)) indicates that upon DNase I treatment, the population of cell‐free DNA and the SSC‐low/SYTO 16‐high subset of DNA^+^ EVs disappeared almost completely. To verify the maintenance of EV structure upon DNase I treatment, immunofluorescent labelling of three classic transmembrane proteins CD9, CD63 and CD81 was performed. Figure S8 indicates that no remarkable change was observed in either the SSC or FL signals before and after DNase I digestion. These results indicate that the DNase I treatment protocol used in the present study facilitated an efficient digestion of external EV‐DNA while maintaining the structure and integrity of EVs. Considering that only DNA inside the EVs can be protected from enzyme digestion, the SSC‐low/SYTO 16‐high subset of DNA^+^ EVs can be identified as EVs with DNA mainly attached to the external surface. To exclude the scenario in which DNA and EVs were forced together/aggregate during ultracentrifugation, we stained the CCCM directly with SYTO 16 and analysed using nFCM. Figure S9 shows that the percentage of DNA^+^ particles was 30.0% for CCCM. After using DNase I to degrade external DNA, the ratio of DNA^+^ population dropped to 6.5%. Meanwhile, by using 1% Triton X‐100 to lyse EVs in CCCM, the event rate of DNA^+^ particles with detectable SSC signal dropped from 2554 to 240. These results indicate that DNA^+^ particles detected in CCCM were mainly EVs, and DNA is naturally distributed on the surface of EVs rather than being forced aggregate on EV surface during ultracentrifugation.

To enable an easy interpretation of the correlation of EV‐DNA with EV size, the SSC intensity was converted to EV size based on the procedure described above (Figure 1(d, e)) and the events of cell‐free DNA were ignored. The bivariate dot‐plots of SYTO 16 FL intensity versus EV size shown in Figure 3(c) (i) and (ii) indicate that among all the events with detectable SSC signal, the percentage of DNA^+^ EVs dropped from 60.6% to 26.0% upon DNase I treatment. The distribution histograms of EV size and SYTO 16 FL intensity for DNA^+^ EVs before and after DNase I treatment are plotted in Figure 3(c) (iii) and (iv). Clearly, for the EV preparation from the CCCM of HCT‐15 cells by ultracentrifugation, the EVs with DNA attached to the external surface were of relatively small size (40–100 nm) and contained more DNA content, and EVs with DNA encapsulated inside were of relatively large size ranging approximately from 80 to 200 nm and contained less DNA content. The median FL intensities of external DNA^+^ EVs and internal DNA^+^ EVs were 2350 and 750, respectively, indicating more DNA localizes to the EV surface than inside the lumen. By using a mixture containing 400, 2000 and 5000 bp of DNA fragments as the external standard, the total DNA length of single EVs was estimated and a large variation from 200 to 550,000 bp was identified (Figure S10).

Although SYTO 16 showed a greater preference for DNA staining, it also labelled RNA slightly (Figure S2). To achieve a highly selective labelling of DNA, ethynyl‐modified dUTP (EdU) was incorporated into the newly synthesized DNA via a metabolic biosynthetic pathway followed by the chemoselective coupling with azide‐AF488 via click chemistry (Figure 3d (i)). Single cell analysis using conventional FCM indicated that EdU was successfully incorporated into the cells and 91.7% of the cells were EdU‐positive (Figure S11). However, due to the small DNA content of single EVs, only 11.3% of the EVs were EdU‐positive (Figure 3(d) (iii)). Nonetheless, this population ratio dropped to 3.0% upon DNase I digestion, and the DNA^+^ EVs that showed a significant decrease were of relatively small size, with relatively large EVs showing almost no change. The replicates and statistical analysis for the data in Figure 3(d) is shown in Figure S12. The EdU incorporation experiment confirmed the observation with SYTO 16 nucleic acid staining that internal DNA was mainly encapsulated inside the lumen by EVs having a relatively large size, while external DNA was mainly associated with the outer membrane of relatively small EVs.

To measure the fragment length of the EV‐DNA, the EV preparation was divided into two aliquots: one was treated with DNase I and the other was kept untreated before DNA isolation. Chip‐based capillary electrophoresis of the DNA extracted from EV preparations without DNase I treatment showed that the majority of the total DNA ranged from 1000 to 50,000 bp (Figure 3(e) (i)). At this stage, no DNase was added and the DNA content comprised both outer membrane‐associated and intravesicular EV‐DNA along with cell‐free DNA that had been co‐isolated with EVs. After using DNase I to digest extravesicular DNA (external EV‐DNA and cell‐free DNA), internal EV‐DNA was found in the short size range of 200–1200 bp (Figure 3(e) (ii), inset). Owing to the extremely low content of internal EV‐DNA, internal DNA was concentrated 10‐fold via speed vacuum lyophilization and analysed in parallel with the unconcentrated sample. Noting that we tried to obtain EV preparations without the contamination of cell‐free DNA as the starting material by using size exclusion chromatography (SEC) or density gradient ultracentrifugation to further purity the EV preparation obtained by ultracentrifugation. Single molecule/particle analysis by nFCM indicates that SEC can hardly separate cell‐free DNA from EVs (Figure S13). Whereas density gradient ultracentrifugation can efficiently separate cell‐free DNA from EVs, EV‐DNA adhered to the outer membrane of EVs was largely detached from the surface owing to the harsh separation process (Figure S14).

### 3.4 In situ differentiation between ssDNA and dsDNA for both external and internal EV‐DNA

**FIGURE 4:**
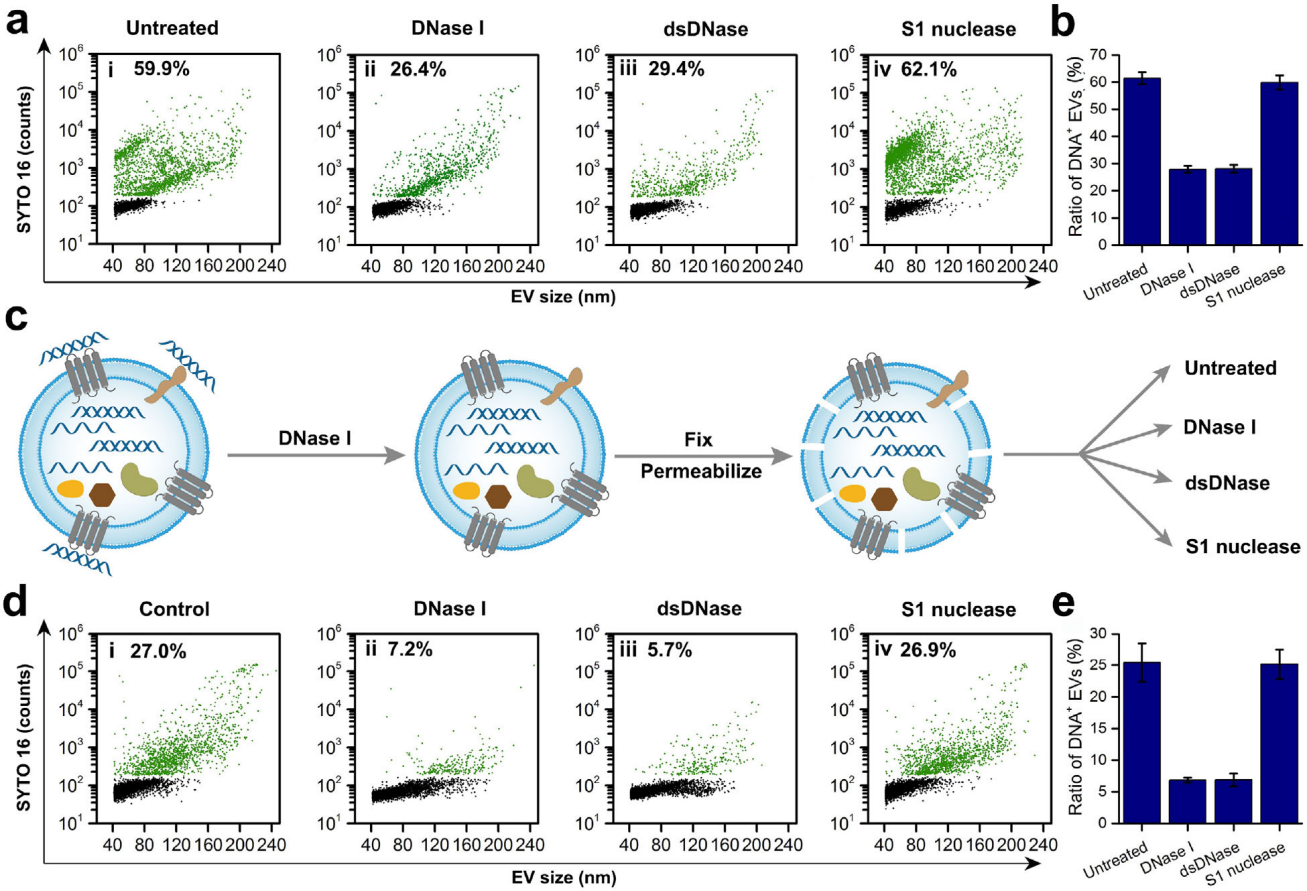
In situ single‐stranded DNA (ssDNA)/double‐stranded DNA (dsDNA) differentiation for both external and internal DNA in EVs (EV‐DNA). (a) Bivariate dot‐plots of SYTO 16 fluorescence versus extracellular vesicles (EV) size for EV preparations untreated (i) or treated with DNase I (ii), dsDNase (iii) or S1 nuclease (iv) before SYTO16 staining. (b) The population ratios of DNA^+^ EVs for EV preparations treated without or with different DNases. (c) The schematic of ssDNA/dsDNA in situ differentiation for internal EV‐DNA. (d) Bivariate dot‐plots of SYTO 16 fluorescence versus EV size for EV preparations untreated (i) or treated with DNase I (ii), dsDNase (iii) or S1 nuclease (iv) after removal of external EV‐DNA and upon EV fixation and permeabilization. (e) The population ratios of DNA^+^ EVs for EV preparations treated without or with different DNases after removal of external EV‐DNA and upon EV fixation and permeabilization. For (b) and (e), three independent experiments were conducted, and data are represented as mean ± s.d. (*n* = 3)

To determine whether external EV‐DNA is ssDNA or dsDNA, EV preparations without or with treatment with DNase I, dsDNase (specifically digest dsDNA) or S1 nuclease (specifically digest ssDNA) were labelled with SYTO 16 and analysed on the nFCM. The functionality and specificity of dsDNase and S1 nuclease were verified using purified lambda DNA and ssDNA oligonucleotides as substrates (Figure S15). Figure 4(a) shows the bivariate dot‐plots of SYTO 16 FL versus EV size for EV preparations treated without or with different DNases for events with detectable SSC signal. The proportion of DNA^+^ EVs decreased significantly from the untreated 59.9% to 26.4% upon DNase I digestion, consistent with the results shown in Figure 3(a, b). Pre‐treatment with dsDNase also resulted in a strong reduction of the DNA^+^ EV ratio to 29.4%, similar to the DNase I treatment. Meanwhile, no remarkable change was observed for EVs treated with S1 nuclease (62.1%) versus the untreated EV sample (59.9%). The results of three replicate experiments are shown in Figure 4(b), implicating that external EV‐DNA is primarily double‐stranded.

Figure 4(c) shows a schematic of the in situ differentiation between ssDNA and dsDNA for internal EV‐DNA. External EV‐DNA was first eradicated using DNase I. Then, the EVs were fixed, permeabilized and treated without or with different DNases. Figure 4(d) (i) shows that after DNase I treatment along with fixation and permeabilization, the percentage of DNA^+^ EV was 27.0%. The comparable population of DNA^+^ EV and EV distribution patterns in Figures 4(d) (i) and 4(a) (ii) indicate that the procedure for EV membrane fixation and permeabilization had negligible effect on the structural integrity of EVs. When internal EV‐DNA was digested with DNase I, the population ratio of DNA^+^ EVs dropped significantly to 7.2% (Figure 4(d) (ii)). When internal EV‐DNA was treated with dsDNase, a remarkable decrease in the events of DNA^+^ EVs (to 5.7%) was observed (iii). Meanwhile, S1 nuclease treatment resulted in almost no decrease in the DNA^+^ EV population (iv). The data from three replicate experiments for each sample type are shown in Figure 4(e). These results indicate that dsDNA is the predominant form of internal EV‐DNA.

### 3.5 Inhibition of exosome secretion reduces the quantity of the relatively small‐sized EVs of which DNA mainly attaches to the external surface

**FIGURE 5:**
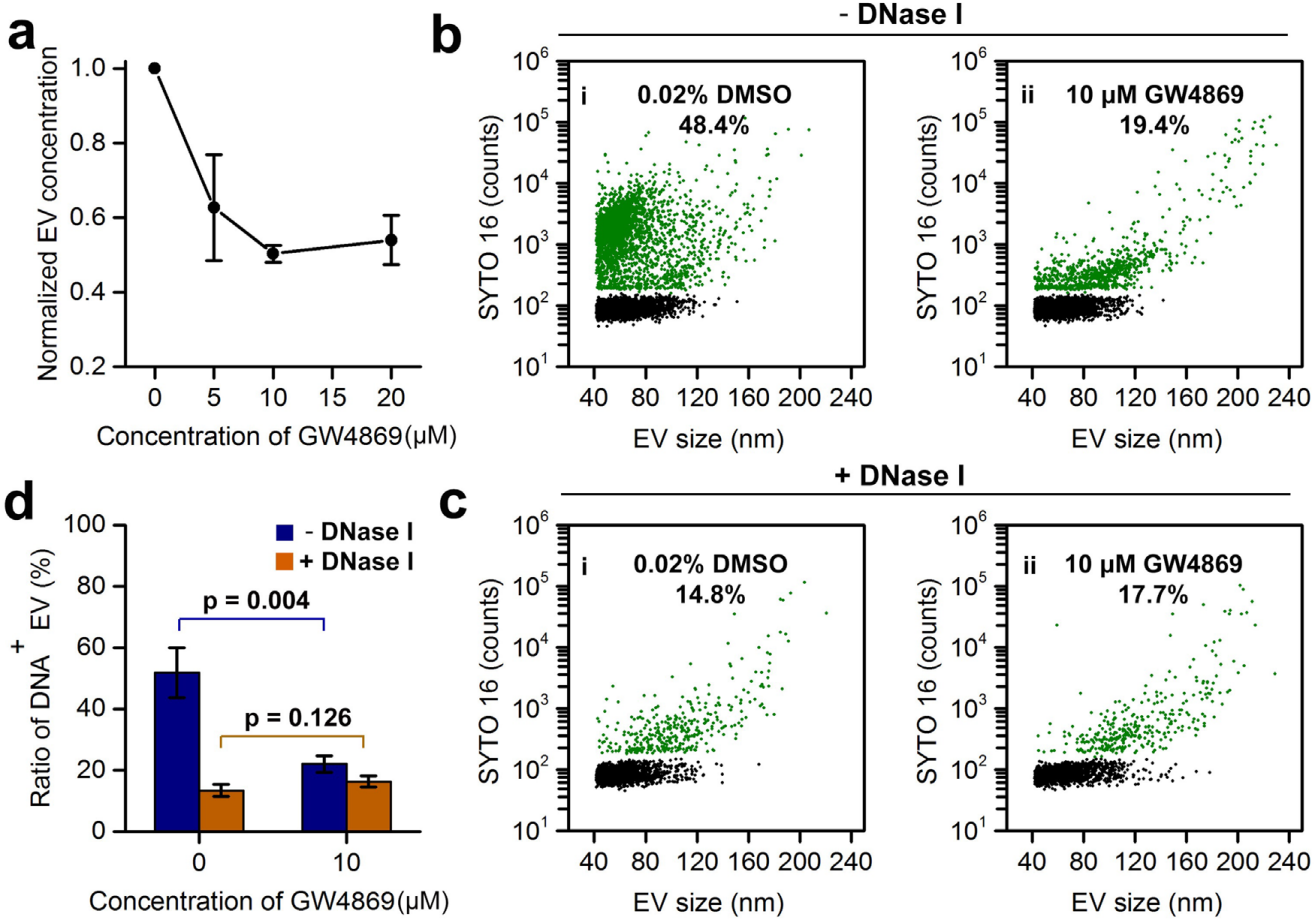
The impact of exosome secretion inhibitor GW4869 on EV‐DNA. (a) Dependence of extracellular vesicles (EV) concentration derived from HCT‐15 cells on the concentration of GW4869. (b, c) Bivariate dot‐plots of SYTO16 fluorescence versus EV size for EVs harvested from the conditioned cell culture medium (CCCM) of HCT‐15 cells without (i) or with 10‐μM GW4869 treatment for 24 h (ii) before (b) and after (c) DNase I treatment. (d) Bar graph of the ratio of DNA^+^ EVs for EVs with or without GW4869 inhibition and before or after DNase digestion. Three independent experiments were conducted, and data are represented as mean ± s.d. (*n* = 3)

Figures 3 and 4 reveal that there seem to exist two populations of EVs, EVs of relatively small size (40–100 nm) mainly carrying long and more DNA fragments attached to the external surface and EVs of relatively large size (80–200 nm) generally having short and less DNA fragments (0.2–2 kbp) encapsulated inside. To investigate whether external and internal EV‐DNA are derived from different biogenesis pathways, GW4869, a specific inhibitor of neutral sphingomyelinase, which has been shown to inhibit exosome secretion (Trajkovic et al., 2008) was used to treat HCT‐15 cells for 24 h. Figure 5(a) shows that with the increase of GW4869 concentration, the quantity of EVs harvested from the CCCM of HCT‐15 cells decreased until reaching a plateau at approximately 50% of the original value obtained at 10‐μM GW4869. Figure 5(b) indicates that the ratio of DNA^+^ EVs decreased from 48.4% of the DMSO control to 19.4% upon 10‐μM GW4869 treatment, and EVs with decreased DNA content were mainly EVs smaller than 100 nm. These results agree with the notion that exosomes are mainly small EVs. DNase I treatment was then performed for EV preparations with or without GW4869 treatment. The comparison of the bivariate dot‐plots of SYTO 16 FL versus EV size shown in Figures 5(b) (i) and 5(c) (i), shows a similar phenomenon to that observed in Figure 3(a,b). For most small‐sized EVs, DNA was mainly attached to the external surface and was degraded upon DNase I treatment; however, for the majority of large‐sized EVs, DNA was mainly enclosed within EVs and was well protected from DNase I digestion. When comparing Figures 5(b) (ii) and 5(c) (ii), no remarkable difference was identified for the population of DNA^+^ EVs before and after DNase I digestion (19.4% vs. 17.7%) for EVs isolated from cells treated with GW4869. These results suggest that when cells were cultured in GW4869, the secretion of exosomes was largely inhibited. As the remaining EVs were relatively large (80–200 nm) and DNA was mainly enclosed inside the lumen, DNase I digestion resulted in a minimal effect on the isolated EVs. This population could be ascribed to microvesicles formed by direct budding from the plasma membrane. The results of three replicate experiments for each treatment are shown in Figure 5(d). When the exosome secretion pathway was inhibited by 10‐μM GW4869, the EV population with DNA attached to the external surface or the population disappear upon DNase I treatment decreased significantly from the original 38.4% (51.8%–13.4%) to 5.7% (22.0%–16.3%).

### 3.6 Histones (H3) are not found in EVs and EV‐DNA is not associated with histone proteins

**FIGURE 6:**
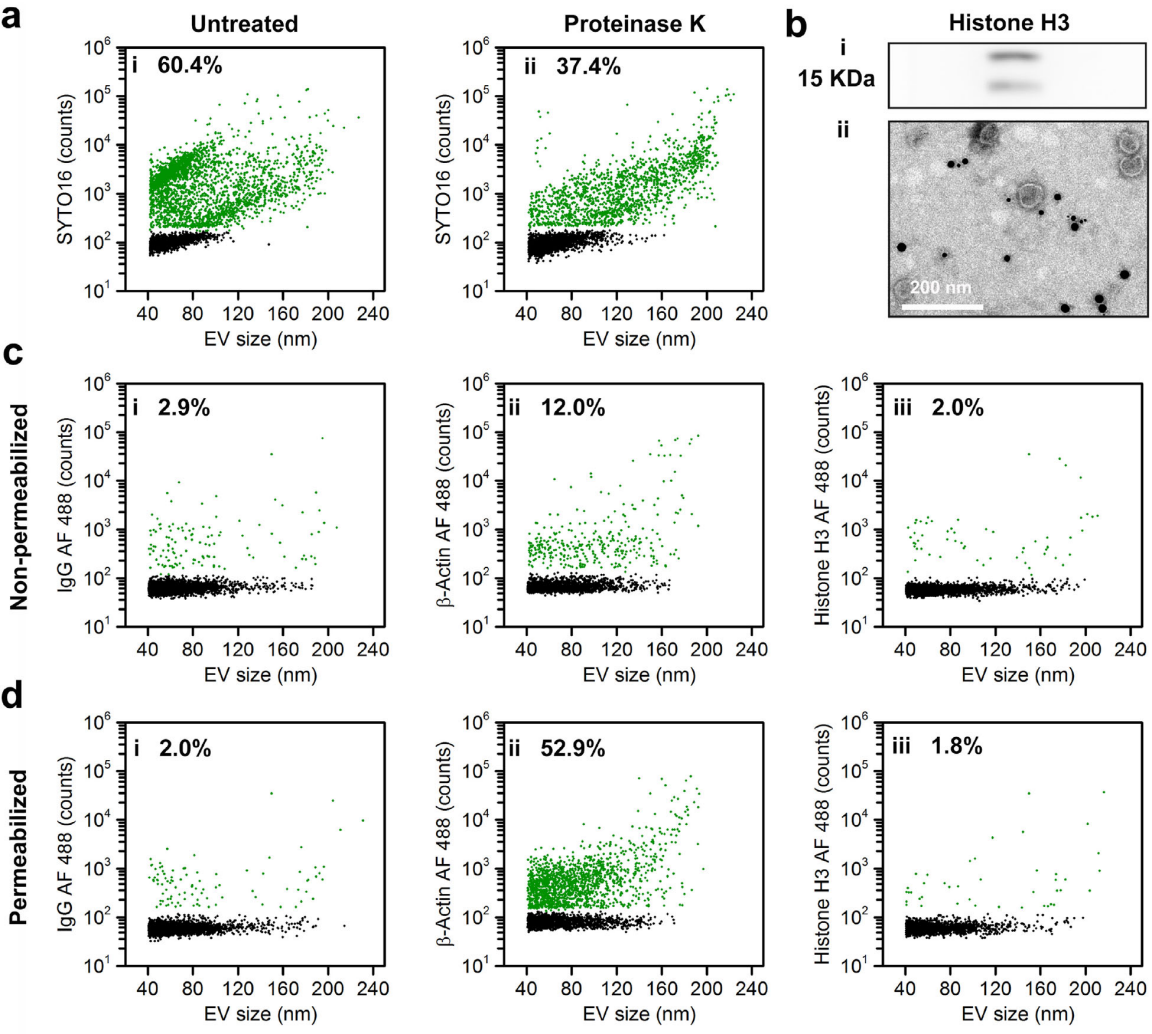
Identification of histone proteins in extracellular vesicles (EV) preparations. (a) Bivariate dot‐plots of SYTO 16 DNA fluorescence versus EV size for EVs without (i) or with (ii) proteinase K treatment. (b) Western blot (i) and immuno‐gold (ii) analysis of histone H3 for EV preparations from HCT‐15 cells. (c) Bivariate dot‐plots of AF 488 green fluorescence versus EV size for non‐permeabilized EVs. The EVs were fluorescently labelled with AF 488‐conjugated mAbs specific to IgG (i), β‐actin (ii) or histone H3 (iii). (d) Bivariate dot‐plots of the AF 488 green fluorescence versus EV size for permeabilized EVs. The EVs were fluorescently labelled with AF 488‐conjugated mAbs specific to IgG (i), β‐actin (ii) or histone H3 (iii)

The data shown above indicate that EVs of relatively small size (<100 nm) mainly contain DNA attached to the external surface. To investigate whether this adhesion occurs through the attachment of membrane proteins, isolated EVs were treated with proteinase K, stained with SYTO 16 and analysed on the nFCM. Figure 6(a) indicates that the ratio of DNA^+^ EVs decreased from the untreated 60.4% to 37.4%, suggesting that 23.0% of EVs had their DNA detached from the EV surface upon proteinase K treatment. Comparing this detachment rate to the 33.5% (61.4%–27.9%, Figure 4(b)) degradation rate of DNase I treatment, it is suggested that most EV‐DNA presented on the external surface is through the attachment to outer membrane proteins. Since DNA‐binding histone proteins have been identified in EV preparations (Jeppesen et al., 2019; Lazaro‐Ibanez et al., 2019; Vagner et al., 2018; Yokoi et al., 2019), present study examined whether there is any relationship between EV‐DNA and histone proteins. Western blot analysis confirmed the presence of histone H3 in EV preparations (Figure 6(b) (i)), and immuno‐gold TEM analysis revealed that histone H3 was mainly associated with small non‐vesicular particles of the EV preparation rather than the external surface of EVs (Figures 6(b) (ii) and S16). To determine whether histones are present on the surface or inside EVs, immunophenotyping was performed by incubating fluorescently labelled antibodies with EVs without or with membrane permeabilization. After fixation with 4% PFA, PBS containing 0.2% Tween 20 (v/v) was used to permeabilize the EV membrane, and β‐actin, a cytoskeleton protein mainly recovered in the EV lumen rather than the EV outer membrane [44,45] served as the positive control. Figures 6(c) (ii) and 6(d) (ii) show that the population ratio of β‐actin^+^ EVs increased from 12.0% to 52.9% after EV membrane permeabilization, demonstrating a good efficiency of EV membrane permeabilization and luminal protein labelling. On the other hand, the measured percentages of histone H3^+^ EVs without and with EV membrane permeabilization were comparable (2.0% vs. 1.8%) (Figures 6(c) (iii) and 6(d) (iii)), close to the levels of their corresponding isotype controls (Figures 6(c) (i) and 6(d) (i)). Moreover, conventional FCM analysis of HCT‐15 cells revealed the presence of histone H3 in the cell, and that the monoclonal antibody used was good for histone H3 labelling (Figure S17). Thus, these results suggest that histones are not found in EVs; however, they are associated with the non‐vesicular particles co‐isolated with EVs. EV‐DNA is thus not associated with histone proteins.

### 3.7 Anti‐cancer drug induces increase in both the population of DNA^+^ EVs and the DNA content in individual EVs

**FIGURE 7:**
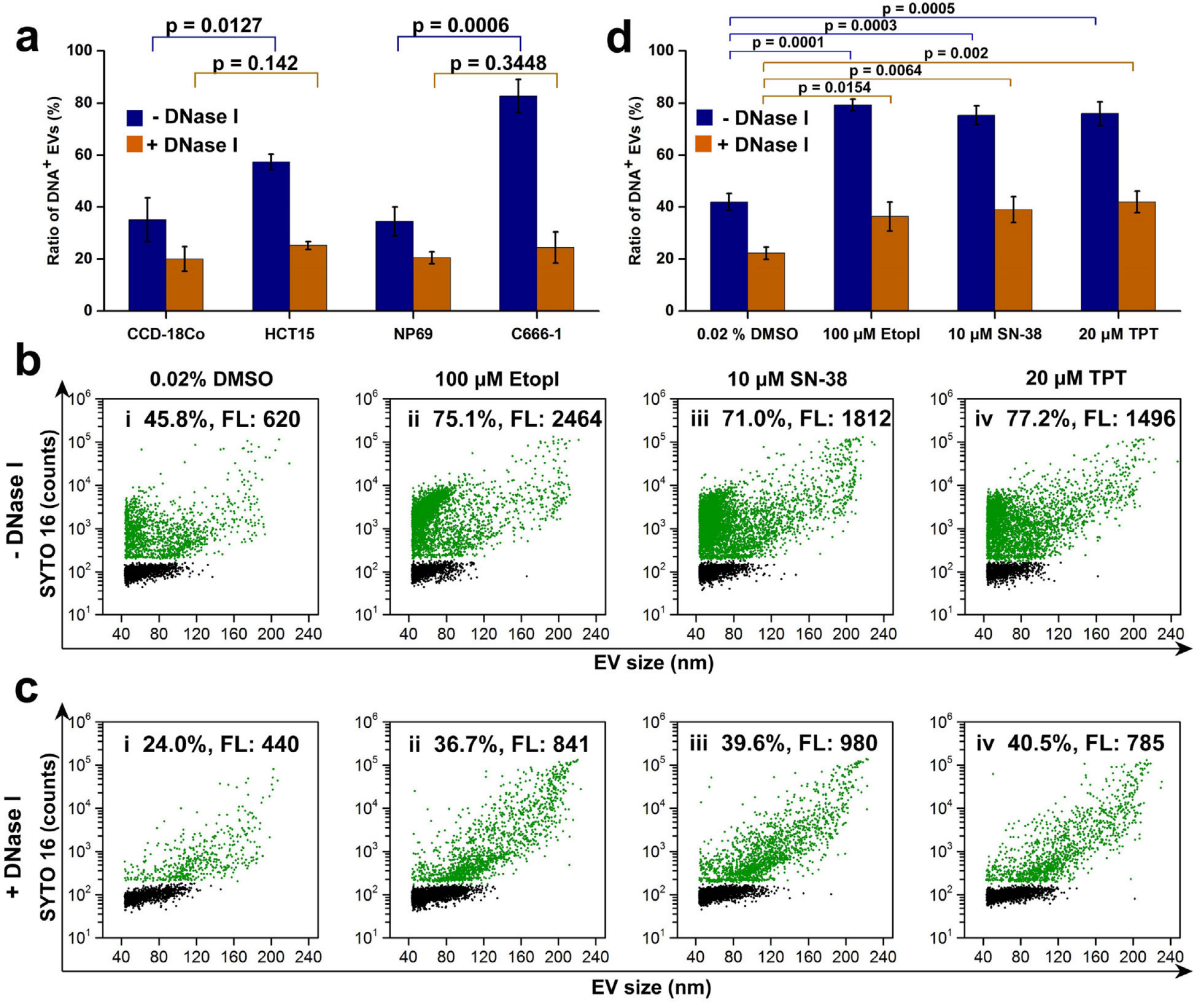
Comparison of DNA in EVs (EV‐DNA) between normal cells and cancer cells along with the study of anticancer drug treatment on EV‐DNA of cancer cells. (a) The population ratios of DNA^+^ extracellular vesicles (EVs) for EV preparations from cancer cell lines and normal cell lines (*n* = 3, mean ± s.d.). (b) Bivariate dot‐plots of SYTO 16 fluorescence versus EV size of EVs for EV preparations from HCT‐15 cells treated with DMSO control (i), etoposide (ii), SN‐38 (iii) or topotecan (iv). (c) Bivariate dot‐plots of SYTO 16 fluorescence versus EV size of DNase I‐treated EVs for EV preparations from HCT‐15 cells treated with DMSO control (i), etoposide (ii), SN‐38 (iii) or topotecan (iv). (d) The population ratios of DNA^+^ EVs for EV preparations from HCT 15 cells treated with DMSO control and anti‐tumour drugs (*n* = 3, mean ± s.d.)

EVs play important roles in maintaining cellular homoeostasis by drawing off harmful cytoplasmic DNA from cells (Lian et al., 2017; Takahashi et al., 2017). It has been reported that EVs secreted by cancer cells carry more DNA fragments than those released by normal cells (Thakur et al., 2014). In this study, EV‐DNA comparison was made for two pairs of normal and cancer cell lines: human colon fibroblast cell line CCD‐18Co and human colorectal cancer cell line HCT‐15, human nasopharyngeal epithelial cell line NP69 and nasopharyngeal carcinoma cell line C666‐1. By comparing the amount of DNA^+^ EVs secreted by normal and cancerous cells, it was found that cancer cells secreted a significantly higher proportion of DNA^+^ EVs (CCD‐18Co, 35.1 ± 8.4%; HCT‐15, 57.3% ± 3.0%; NP69, 34.4 ± 5.6% and C666‐1, 82.7 ± 6.4%) (Figures 7(a) and S18(a)). Upon DNase I treatment to eradicate external EV‐DNA, the percentage of DNA^+^ EVs was comparable between normal cells and cancer cells (CCD‐18Co, 20.1 ± 4.6%; HCT‐15, 25.2% ± 1.5%; NP69, 20.5 ± 2.2% and C666‐1, 24.5 ± 6.0%) (Figures 7(a) and S18(b)). These data suggest that due to the inherent genomic instability, cancer cells secrete more DNA^+^ EVs than normal cells, and this discrepancy is mainly due to the difference in the proportions of external DNA^+^ EVs.

Increasing evidence indicates that anti‐tumour drugs induce considerable chromosomal instability and cell senescence. As a result, cancer cells alter their EV secretion profile by releasing a large number of EVs carrying more DNA content to maintain cell homoeostasis (Choi et al., 2019; Kitai et al., 2017; Lian et al., 2017). To examine EV‐DNA changes at the single‐EV level, HCT‐15 cells were treated with genotoxic drug etoposide (an inhibitor of topoisomerase II) (100 μM), SN‐38 (an inhibitor of topoisomerase I) (10 μM), or topotecan (an inhibitor of topoisomerase I) (20 μM). Upon treatment with either drug, the population of EVs containing DNA increased significantly from 45.8% for the control (0.02% DMSO) to 75.1%, 71.0% and 77.2% for etoposide, SN‐38 and topotecan, respectively (Figure 7(b)). Moreover, the median FL intensity, that corresponding to DNA content in individual EVs, also increased markedly from 620 for the control to 2464, 1812 and 1496 for etoposide, SN‐38 and topotecan, respectively. These results suggest that both the number of DNA^+^ EVs and the DNA quantity in single EVs increased following anticancer drug treatment. To investigate the change in internal EV‐DNA upon drug treatment, DNase I was used to digest external EV‐DNA before SYTO 16 staining. Figure 7(c) reveals a significant increase in the population of internal DNA^+^ EVs upon treatment with either drug (0.02% DMSO, 24.0%; etoposide, 36.7%; SN‐38, 39.6% and topotecan, 40.5%). Meanwhile, the median FL intensity also increased remarkably (0.02% DMSO, 440; etoposide, 841; SN‐38, 980 and topotecan, 785). The data from three replicate experiments for each treatment are shown in Figure 7(d). The population ratios of DNA^+^ EV were 42.0 ± 3.3%, 79.2% ± 2.3%, 75.3 ± 3.6% and 75.9 ± 4.6% for 0.02% DMSO, 100‐μM etoposide, 10‐μM SN‐38, and 20‐μM topotecan, respectively. After DNase I treatment, the population ratios of internal DNA^+^ EVs were 22.3 ± 2.4%, 36.4% ±  5.5%, 38.9 ± 5.0% and 41.9 ± 4.1% for 0.02% DMSO, 100‐μM etoposide, 10‐μM SN‐38, and 20‐μM topotecan, respectively.

## 4 DISCUSSION

The growing interest and the recognized relevance of EV‐DNA to disease call for an in‐depth understanding of how DNA is associated with different EV subsets and their respective functions. EV‐DNA analysis at the single‐vesicle level can reveal the large intrinsic heterogeneity among individual EVs and overcome the ambiguity in interpreting data obtained from ensemble‐averaged measurements. Using the cutting‐edge nano‐flow cytometry to examine SSC and FL signal concurrence of single particles, the populations of FL only (cell‐free DNA), SSC only (debris or DNA^–^ EVs), and FL with concurrent SSC (DNA^+^ EVs) can be clearly differentiated from each other without physical separation of these constituents. Present study identified an abundant presence of cell‐free DNA (naked DNA or DNA associated with non‐vesicular entities of which the SSC was undetectable by nFCM) in EV preparation from CCCM by ultracentrifugation. It is also revealed for the first time that SEC cannot efficiently separate cell‐free DNA from EVs and density gradient ultracentrifugation results in an almost complete eradication of external EV‐DNA. Hence, great efforts must be made to ensure sufficient purification prior to the down‐stream characterization of EV‐DNA by gel/chip electrophoresis, PCR or DNA sequencing as these techniques cannot differentiate cell‐free DNA from EV‐DNA. Importantly, by converting the SSC intensity to particle size via the calibration curve built by monodisperse silica nanoparticles of known size and upon refractive index correction based on the Mie theory (Tian et al., 2020; Tian et al., 2018), the EV‐DNA content can be directly correlated with the EV size.

Regarding the DNA content distribution in EVs, present study found that approximately 61% of the EVs secreted by HCT‐15 cells carry DNA, and the DNA quantity in single EVs exhibited three orders of magnitude variation in terms of FL intensity (Figure 4(b)). Of particular note, two distinct EV subsets with respect to EV size and DNA localization were revealed, namely EVs with a relatively small size (<100 nm), mainly having DNA attached to the external surface and EVs with a relatively large size (80−200 nm), mainly having DNA localized within the lumen. It would be very interesting to identify which population is exosomes and which population is microvesicles. Apparently, more studies are needed to solve this puzzle. Although the populations of external DNA^+^ EVs and internal DNA^+^ EVs were approximately 33% and 28% of the total EVs, respectively, the median FL intensity of external DNA^+^ EVs was approximately 3‐fold higher than that of internal DNA^+^ EVs. This finding is consistent with a recent study in glioblastoma cell‐derived EVs. By using direct stochastic optical reconstruction microscopy to quantify the number of DNA fragment localizations per EV, it was found that the amount of DNA on the surface of EVs was ∼3‐fold higher than the internal amount (Maire et al., 2021). Although it has been well‐recognized that EVs carry DNA, knowledge regarding their identity as dsDNA or ssDNA is still under debate. Using two orthogonal methods of enzymatic digestion and atomic force microscopy, Thakur et al. demonstrated that cancer cell‐derived EVs mainly carry dsDNA (Thakur et al., 2014). Others reported that EV‐DNA mainly contains ssDNA (Balaj et al., 2011; Lazaro‐Ibanez et al., 2019), yet the relative abundance of ssDNA may also be due to the denaturation of dsDNA during the harsh DNA extraction process (Vagner et al., 2018). In present study, dsDNA/ssDNA analysis via in situ enzymatic treatment with DNase I, dsDNase and S1 nuclease before and after EV membrane permeabilization and fixation showed that dsDNA is the predominant form in both internal and external EV‐DNA. By using 10‐μM GW4869 to inhibit exosome secretion, the ratio of DNA^+^ EV decreased from 51.7 ± 8.2% to 22.0 ± 2.7%, and this reduction was mainly due to a diminished secretion of small‐sized EVs (<100 nm) with DNA associated with the external surface (Figure 5(b,d)). Based on these results, it could be suggested that exosomes mainly carry external DNA, while microvesicles contain DNA enclosed inside the lumen. Inward budding inside the late endosomes could be responsible for the outer surface of the exosome being exposed to the endosomal DNA, which results in DNA attaching to the external surface of exosomes (Saari et al., 2020).

It has been suspected that DNA‐binding histones can potentially mediate DNA transfer across cells (Lazaro‐Ibanez et al., 2019; Vagner et al., 2018). Our proteinase K treatment results suggest that the mechanism by which EV‐DNA attaches to the external surface was indeed through membrane protein binding; however, single EV immunophenotyping indicated that histone H3 was not found on the outer membrane nor in the lumen of EVs. Moreover, the immuno‐gold TEM experiment confirmed that instead of being associated with EVs, histone H3 was mainly localized on small non‐vesicular entities co‐isolated with EVs. Thus, the observed presence of histone proteins in EV preparations could be due to the contamination of non‐vesicular entities (Lazaro‐Ibanez et al., 2019; Vagner et al., 2018). Present study supports the perception that histones are not associated with exosomes or other types of small EVs (Jeppesen et al., 2019). Nevertheless, contrary to this report, which stated that small EVs do not contain DNA (Jeppesen et al., 2019), we observed EV‐DNA localized on the surface and inside the lumen of EVs. Moreover, to the best of our knowledge, this is the first report of luminal protein phenotyping of EVs using flow cytometry. The EV membrane permeabilization protocol developed in the present study provides an efficient approach to study the composition and function of EV luminal proteins.

By comparing two pairs of normal and cancer cell lines, present study found that the population ratio of DNA^+^ EVs was higher for cancer cells, which agreed well with the literature report (Thakur et al., 2014). Moreover, it was also identified that this ratio difference was solely due to an elevated EV population with DNA adhered to the external surface as DNase I treatment resulted in a relatively consistent level of DNA^+^ EV ratio for both the normal and cancer cells. When the cancer cells were treated with anti‐tumour drugs such as etoposide, SN‐38 and topotecan, a significant increase in both the population ratio of DNA^+^ EVs and the DNA content in single EVs was observed. This study also identified for the first time that both the external DNA^+^ EV and internal DNA^+^ EVs contributed to the increased secretion of DNA^+^ EVs, implicating the important role that EVs play in maintaining cell homoeostasis. It has been proposed that inhibition of EV release could enhance the efficacy of cancer chemotherapy by preventing the removal of chemotherapeutic drugs from cells through EV transport (Jorfi et al., 2015; Koch et al., 2016; Kosgodage et al., 2018). Present study suggests that the efficient role of EV secretion inhibitors in promoting the efficacy of anti‐tumour drugs could be partially attributed to DNA accumulation in the cytoplasm, which can induce apoptosis.

In summary, by enabling simultaneous light scattering (down to 40‐nm single EVs) and FL detection (down to 200‐bp single DNA fragments) of single particles, nFCM offers an unprecedented perspective on EV‐DNA. It was revealed that: (1) naked DNA or DNA associated with non‐vesicular entities is abundantly presented in EV samples prepared from cell culture medium by ultracentrifugation; (2) the quantity of EV‐DNA in individual EVs exhibits significant heterogeneity and the population of DNA^+^ EVs varies from 30% to 80% depending on the cell type; (3) external EV‐DNA is mainly localized on relatively small size EVs (e.g. <100 nm for HCT‐15 cell line) and the secretion of external DNA^+^ EVs can be significantly reduced by exosome secretion pathway inhibition; (4) internal EV‐DNA is mainly packaged inside the lumen of relatively large EVs (e.g. 80–200 nm for HCT‐15 cell line); (5) dsDNA is the predominant form of both the external and internal EV‐DNA; (6) histones (H3) are not found in EVs and EV‐DNA is not associated with histone proteins and (7) genotoxic drug induces more release of DNA^+^ EVs, and the number of both external DNA^+^ EVs and internal DNA^+^ EVs as well as the DNA content in single EVs increase significantly. Although the present study provides conclusive determination of several important EV‐DNA features, more studies are needed to clarify the mechanisms underlying EV DNA packaging and its functional relevance to normal and cancer cells.

## CONFLICT OF INTEREST

X.Y. declares competing financial interests as a cofounder of NanoFCM Inc., a company committed to commercializing the nano‐flow cytometry (nFCM) technology.

## Supporting information

Supporting information.

Supporting information.

Supporting information.
