# Supplementary material for: Analysis of extracellular vesicle DNA at the single‐vesicle level by nano‐flow cytometry
Source: J Extracell Vesicles. 2022 Apr 4;11(4):e12206. doi: 10.1002/jev2.12206 (PMC8977970; doi:10.1002/jev2.12206)
Supplement: Supplementary file 1 — Supporting information. [file JEV2-11-e12206-s002.pdf]

Supplementary Material

Analysis of Extracellular Vesicle DNA at the Single-Vesicle Level by Nano-Flow Cytometry

Haisheng Liu, Ye Tian, Chengfeng Xue, Qian Niu, Chen Chen and Xiaomei Yan*

Department of Chemical Biology, MOE Key Laboratory of Spectrochemical Analysis & Instrumentation, Key Laboratory for Chemical Biology of Fujian Province, Collaborative Innovation Center of Chemistry for Energy Materials, College of Chemistry and Chemical Engineering, Xiamen University, Xiamen 361005, People's Republic of China

*To whom correspondence should be addressed. E-mail: xmyan@xmu.edu.cn

This Supplementary Material contains Figures S1-S18.

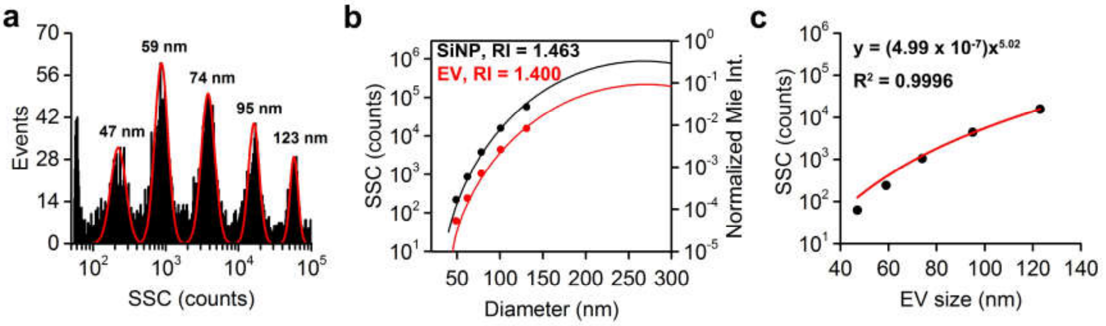

Figure S1. (a) SSC distribution histogram of a mixture of monodisperse SiNPs of five different diameters ranging between 47 and 123 nm, and fit to a sum of Gaussian peaks. (b) Measured (black symbol) and Mie theory calculated (black line) SSC intensity versus diameter for SiNPs along with the predicted (red symbol) and Mie theory calculated (red line) SSC intensity versus diameter for EVs. Mie calculation are in excellent agreement with the data. (c) Plot of the calibration curve between the refractive index corrected-intensity of Gaussian-fitted SiNPs SSC signal and the EV particle size.

Note: Simulation of side-scattered light detected by the nFCM system was performed with MiePlot v4.6 (a computer program for scattering of light from a single sphere using Mie theory and the Debye series) and Origin 8.6 software (Anal. Chem. 2018, 90, 12768−12775). The maximum half angle (α_{max}) of the cone of light that can enter the objective lens is defined as NA = $n_{\mathrm{m}}\sin\alpha_{\mathrm{max}}$, where NA is the numerical aperture of the objective and n_{m} is the refractive index (RI) of surrounding medium. In the present study, the NA of objective lens was 0.55 and n_m was 1.332. Therefore, the calculated α_{max} is 24°. The sheath flow was ultrapure water, and 1.332 was used as the refractive index of the medium. Considering the refractive index difference between SiNPs (1.463) and EVs (1.400) at 488 nm excitation, the intensity ratio between light scattered by a SiNP to that of an EV of the same particle size was calculated based on the Mie theory for every size of the SiNP standard. Then the predicted SSC intensity for an EV of the same particle size of SiNP was obtained by dividing the SSC intensity of the SiNP by the intensity ratio. The calibration curve of SSC intensity of EVs versus diameter was constructed based on the measured SiNPs data and upon refractive index correction (c).

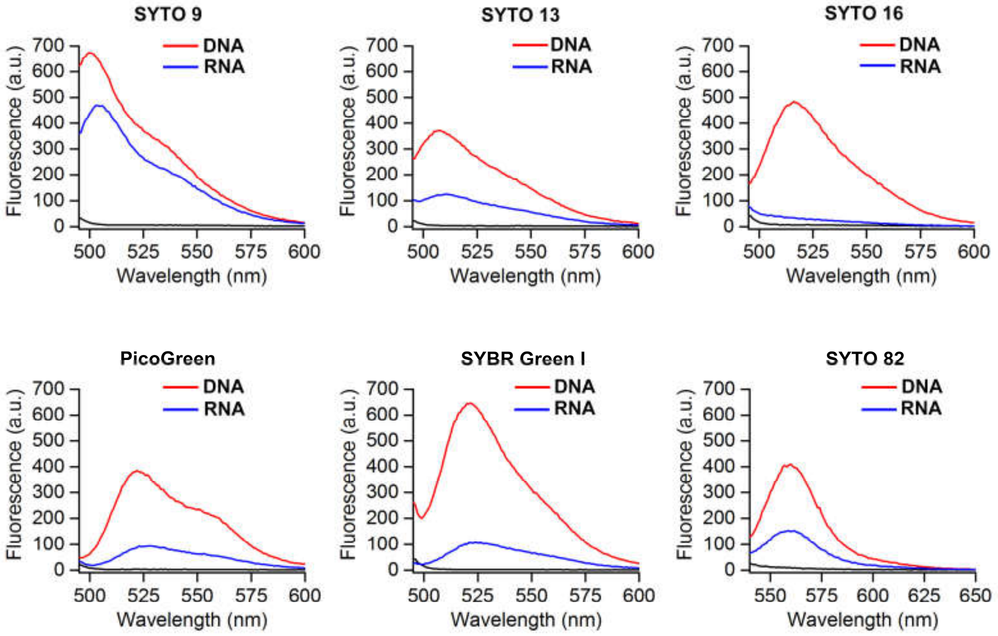

Figure S2. Fluorescence emission spectra of SYTO 9, SYTO 13, SYTO 16, PicoGreen, SYBR Green I, and SYTO 82 without (black line) or with binding to DNA (red line) or RNA (blue line). The concentrations of RNA and DNA were both 5 μg/mL.

Note: All the nucleic acid dyes were purchased from Molecular Probes. Lambda DNA (Promega, D150A) or ribosomal RNA (Thermo Fisher, R11490, component C) with concentration of 5 μg/mL each was incubated with 6 μM SYTO dye or 1× PicoGreen or SYBR Green I for 20 min at 37°C. Fluorescence emission spectra were measured on a Spectrofluorometer (F-5301Pc, Shimadzu) with excitation at 488 nm for SYTO 9, SYTO 13, SYTO 16, PicoGreen, and SYBR Green I and with excitation at 532 nm for SYTO 82.

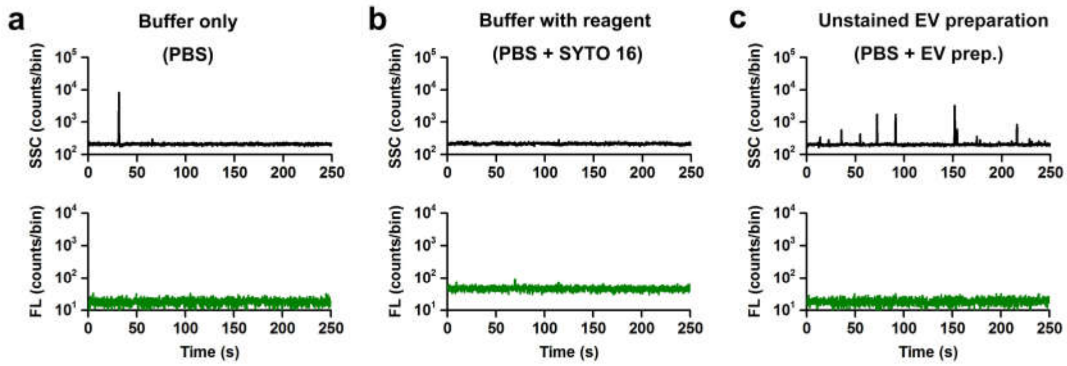

Figure S3. nFCM analysis of the assay controls. Representative SSC and FL burst traces for (a) buffer only, (b) buffer with reagent, and (c) unstained EV preparation.

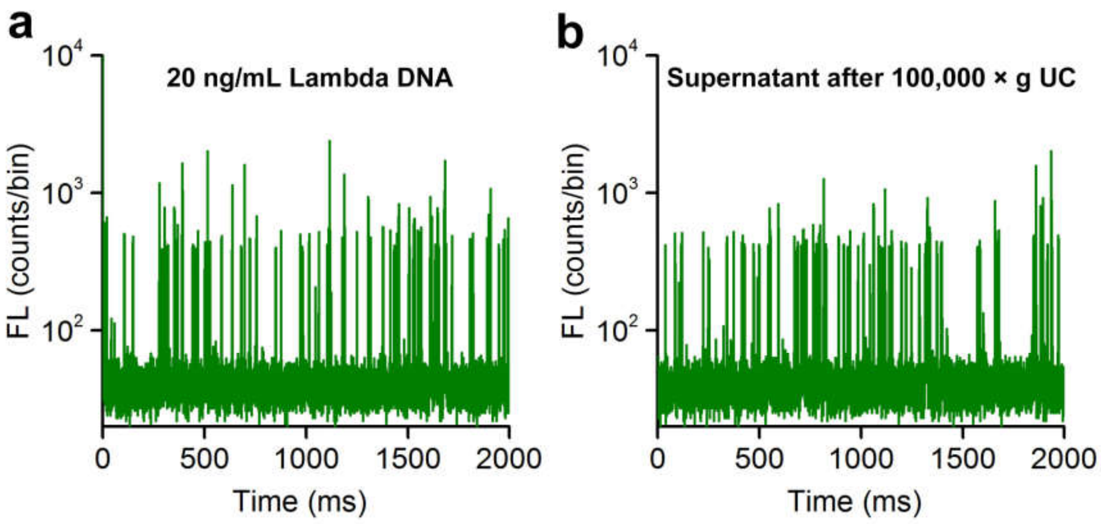

Figure S4. Representative fluorescence burst traces of SYTO 16-stained lambda DNA before (a) and after (b) ultracentrifugation at 100,000 × g for 17 min at 4°C. 7478 and 7575 events were detected in 1 min by nFCM before and after ultracentrifugation, respectively.

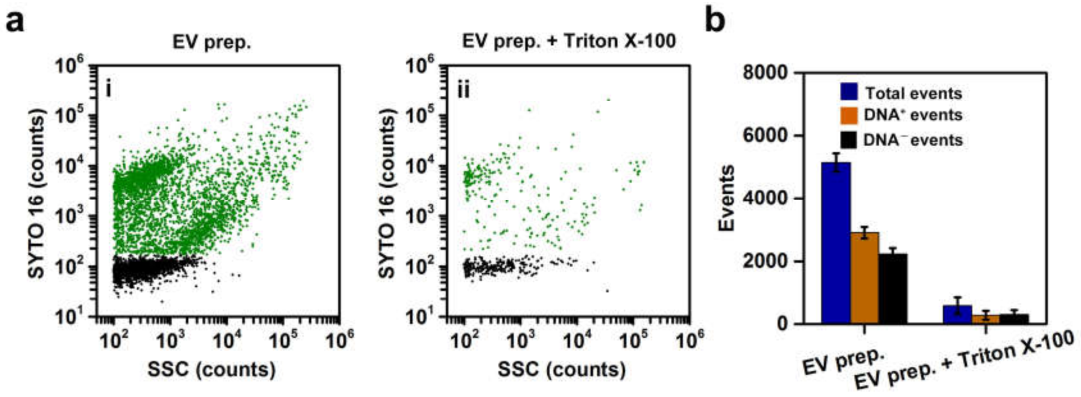

Figure S5. nFCM analysis of EV sample prepared from CCCM of HCT-15 cells by ultracentrifugation without or with Triton X-100 treatment. (a) Bivariate dot-plot of SYTO16 fluorescence versus SSC intensity for an EV preparation before (i) and after 1% Triton X-100 treatment (ii). (b) Bar graphs of the total events, DNA^+ events, and DNA^- events detected in 1 min for EV preparations before and after Triton X-100 treatment. Data are represented as mean ± s.d. (n = 3). Note that SYTO16 staining was carried out after detergent treatment, and the events without detectable SSC signal were ignored.

Note: The threshold levels for both the peak height (a digital discriminator level set to 3 times the standard deviation of the background) and the peak width of 0.2 ms and 0.3 ms were set as the criteria for burst (or peak) identification of SSC and FL signal, respectively. For each burst that satisfied the criteria, the integrated number of photons (background subtracted) was stored as the burst area for the histogram or dot-plot construction. According to the criteria, the FL burst area for each identified fluorescent event is calculated by integrating the number of photons detected in its duration (peak width), i.e. ≥ 0.3 ms (3 bins). For those events that only have SSC signal without FL, their fluorescence burst area was calculated by integrating the number of photons detected within 0.2 ms (2 bins) from the starting point of the SSC burst. Therefore, there exist a distinct discontinuity between the “positive” (green-colored events) and “negative” (black colored events) subsets.

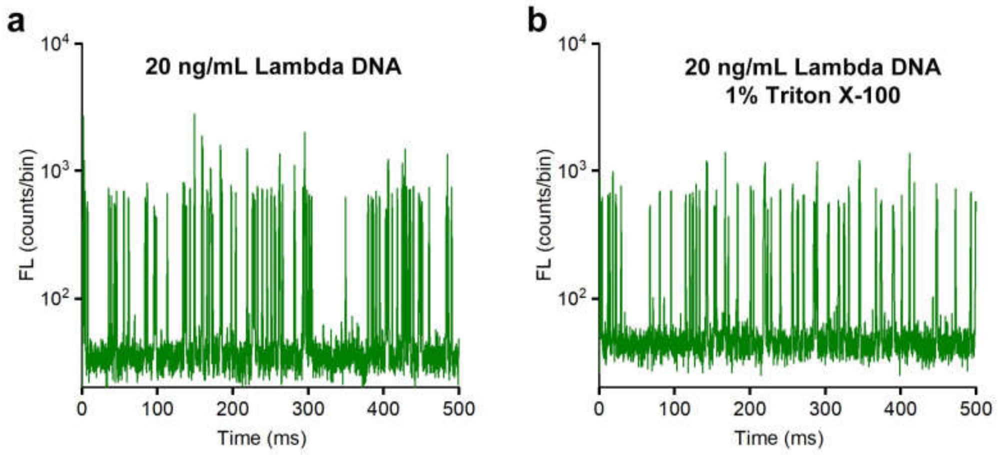

Figure S6. Representative fluorescence burst traces of SYTO 16-stained lambda DNA before (a) and after (b) 1% Triton X-100 treatment for 30 min at 4°C. 8003 and 7280 events were detected in 1 min by nFCM before and after Triton X-100 treatment, respectively.

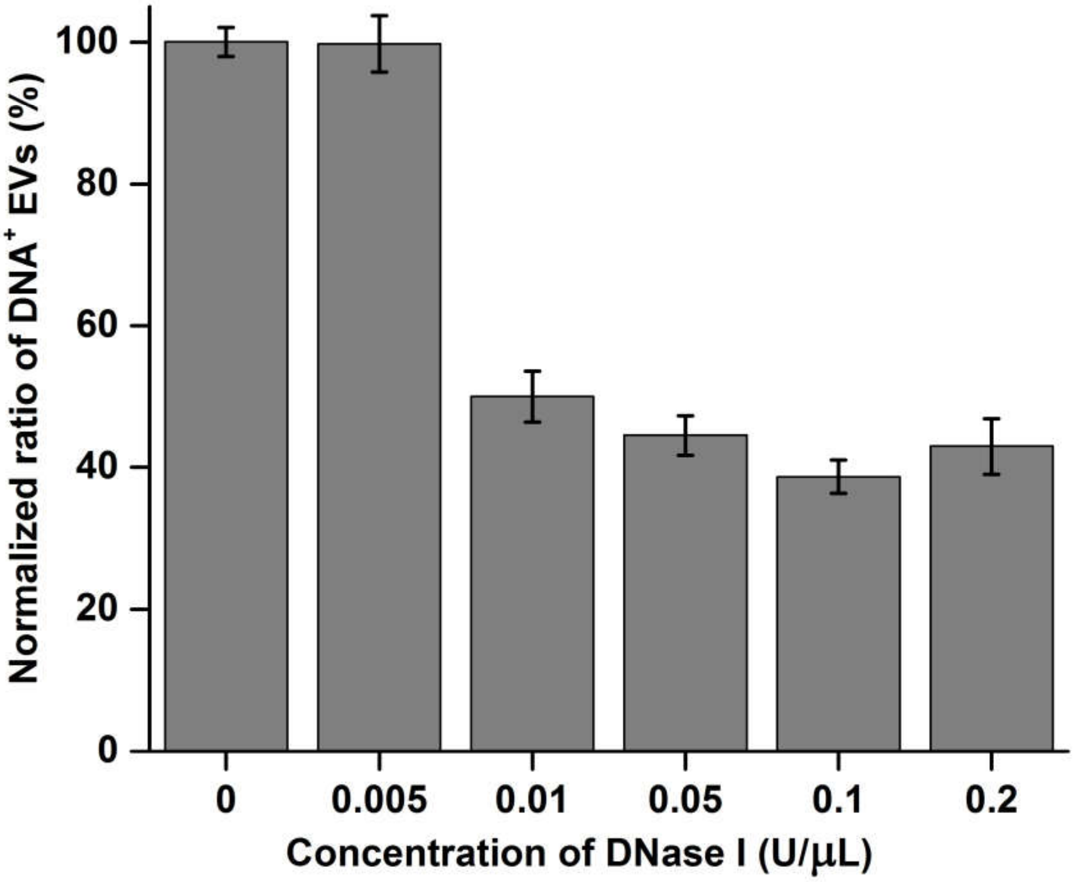

Figure S7. Optimization of the concentration of DNase I. EV preparations were treated with different concentrations of DNase I for 30 min at 37°C, and data are represented as mean ± s.d. (n = 3).

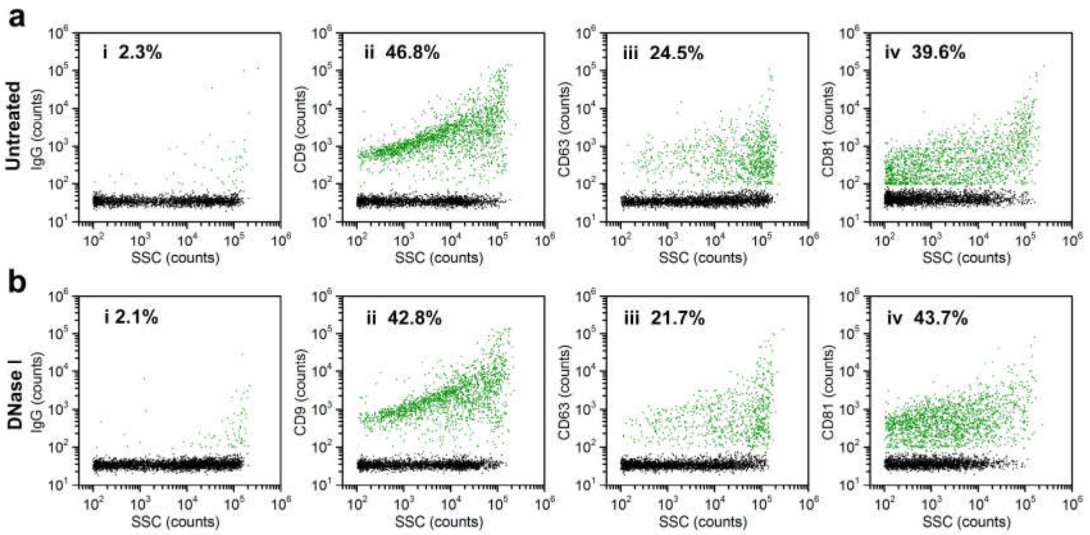

Figure S8. Immunofluorescent analysis of tetraspanin proteins (CD9, CD63, and CD81) for an EV preparation from CCCM of HCT-15 cells by ultracentrifugation without (a) or with (b) DNase I treatment. EVs were stained with phycoerythrin (PE)-conjugated monoclonal antibodies specific to CD9, CD63, or CD81. PE-conjugated IgG was used as the isotype control. The events without detectable SSC signal were ignored.

Note: This measurement was conducted by using 532 nm laser as the excitation source. Two single-photon counting avalanche photodiodes (APDs) were used for the simultaneous detection of side scatter (SSC, bandpass filter: FF01− 524/24) and orange fluorescence (bandpass filter: FF01− 579/34) of individual particles/EVs, respectively.

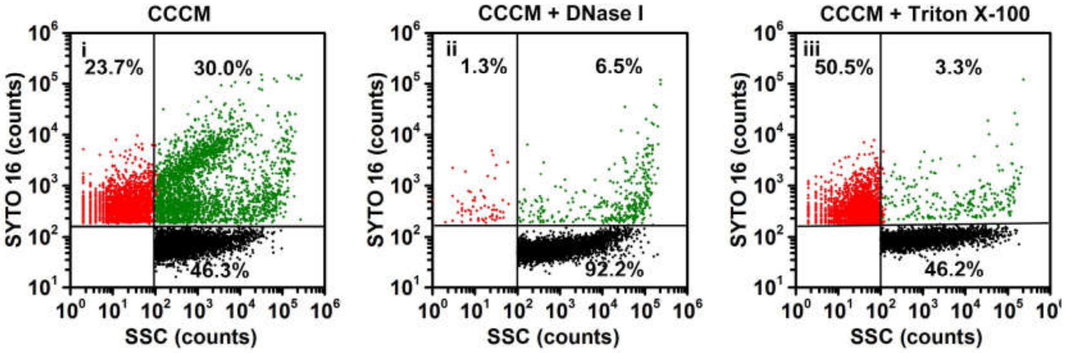

Figure S9. nFCM Analysis of CCCM of HCT-15 cells upon SYTO 16 staining. (a) Bivariate dot-plot of SYTO16 fluorescence versus SSC intensity for CCCM with no treatment (i), with DNase I digestion (ii), or with Triton X-100 lysis (iii). Note that SYTO16 staining was carried out after enzyme or detergent treatment.

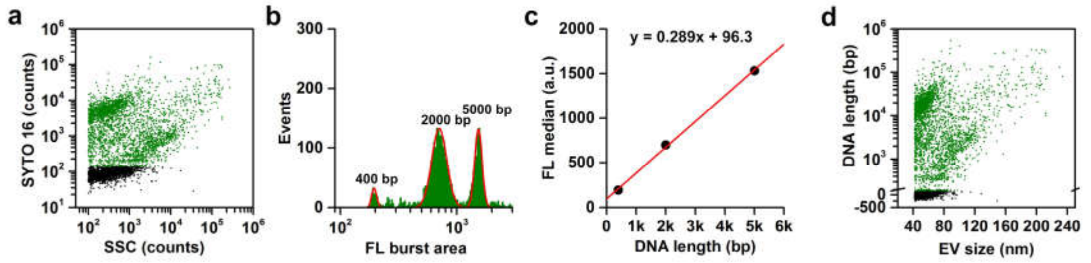

Figure S10. nFCM measurement of DNA content of single EVs in the unit of base pairs by using external DNA standard approach. (a) The bivariate dot-plot of SYTO16 fluorescence versus SSC for the EV preparation from the CCCM of HCT-15 cells by ultracentrifugation. (b) FL burst area distribution histogram for the DNA standard with three different fragment lengths upon SYTO 16 staining. (c) The correlation of the centroids of the burst areas obtained from the fitted Gaussian curve (red line in plot b) with known DNA fragment lengths. (d) The bivariate dot-plot of DNA fragment length in base pairs versus EV size upon converting the fluorescence intensity to base pairs via the calibration curve obtained in plot c and converting SSC intensity to EV size via the calibration curve built by monodispersed silica nanoparticles of know size and upon refractive index correction based on the Mie theory.

Note: The data shown above can only provide an estimation of the total DNA length of single EVs owing to the different staining efficiency between EV-DNA and DNA standard. It may be impractical to accurately convert the FL intensity measured by nFCM to base pairs owing to the following reasons. 1) The success of DNA fragment sizing by flow cytometry relies upon precise, stoichiometric staining of the individual DNA fragments with fluorescent dyes. The longer the DNA fragment, the more the intercalative dye molecules bound and thus the brighter the fluorescence signal. Quantification of total fluorescence emitted by individual DNA fragments as they pass through the laser beam yields the fragment size. However, the staining efficiency of DNA fragment (how many of the intercalative binding sites offered by the DNA fragment are occupied by the intercalative dye molecules) is highly dependent on the DNA concentration, the dye concentration, and the dissociation constant of intercalation (Anal. Chem. 2005, 77, 3554-3562; Anal. Biochem. 2000, 286, 138-148). Thus, for an accurate measurement of DNA fragment

length, DNA standard of known base pairs needs to be added to the sample as an internal standard. 2) For the EV isolate, cell-free DNA exists in great abundance and varies in a wide size range. Thus, it is difficult to identify the peaks of DNA standard spiked in. 3) Even though the peaks of DNA standard can be identified and the intercalating dye is membrane permeable, it is not sure whether the staining condition inside the lumen of EVs is identical to that outside of EVs. 4) The staining efficiency could be different for DNA free in solution or bound to protein complex (e.g. attached to the EV outer membrane or non-vesicular entities).

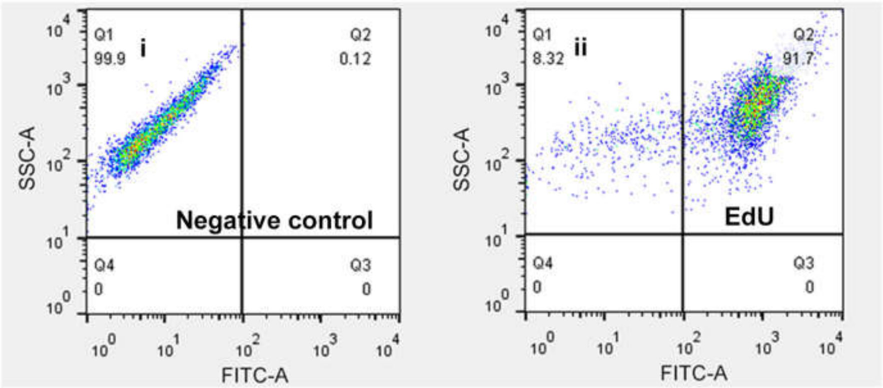

Figure S11. Single cell analysis of HCT-15 cells cultured with 0.02% DMSO (i, negative control) or with the addition of ethynyl-modified dUTP (EdU) (ii) and then labeled with azide-AF488. A BD FACSAria IIIu cytometer with 488 nm excitation was used for the analysis, and the FITC channel (530/30 nm bandpass filter) was used for the fluorescence detection of AF488.

Note: A Click-iT™ Plus EdU Alexa Fluor™ 488 Flow Cytometry Assay Kit (Thermo Fisher, C10632) was used in the present study. The reagents were prepared according to the manufacturer's instructions. Firstly, 0.6 μL of 10 mM EdU in DMSO or 0.6 μL DMSO (negative control) were added to 3 mL cell culture medium. The HCT-15 cells were cultured with these media at 37°C for 72 hours. Then, about 1 × 10^6 cells were collected and centrifuged at 800 × g for 5 min at 4°C. Cells were resuspended in 100 μL 4% PFA and incubated at room temperature for 30 min. The PFA was washed away with 1 mL PBS by ultracentrifugation at 800 × g for 5 min at 4°C. Subsequently, cells were resuspended in 100 μL of 1× saponin-based permeabilization and wash reagent (component E), incubated for 15 min at room temperature. Lastly, 500 μL click-iT reaction cocktail with Azide-AF488, copper protectant, and buffer additive mixed in PBS was added to the cells. The mixture was incubated for 30 min at room temperature and protected from light. After two washes by ultracentrifugation at 800 g for 5 min 4°C with 1 mL of 1× saponin-based permeabilization and wash reagent, the sample was resuspended in 500 μL PBS for BD FACSAria IIIu cytometer analysis.

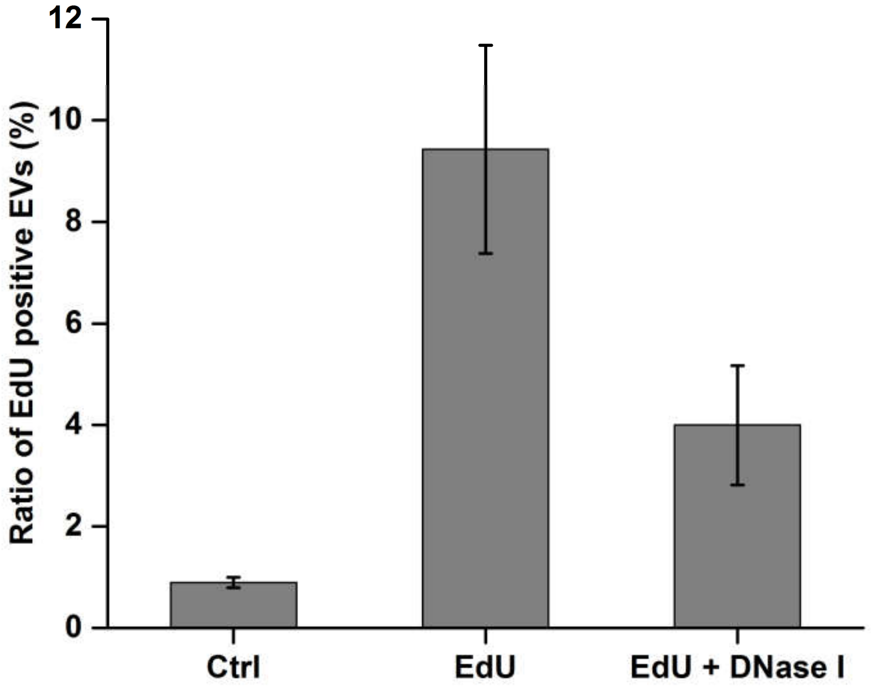

Figure S12. The population ratios of EdU positive EVs for EVs isolated upon ultracentrifugation from the CCCM of HCT-15 cells cultured with 0.02% DMSO (reagent control), 20 μM EdU, or 20 μM EdU and upon DNase I treatment. Data are represented as mean ± s.d. (n = 3).

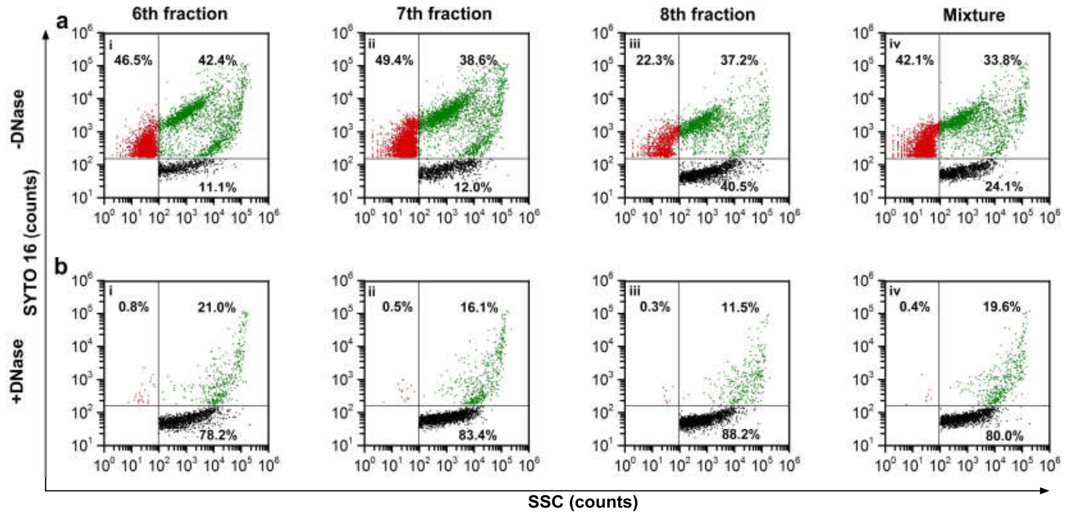

Figure. S13. nFCM examination of the effectiveness of SEC in separating cell-free DNA from EVs for the EV preparation obtained from the CCCM of HCT-15 cells by ultracentrifugation. (a,b) Bivariate dot-plots of SYTO16 fluorescence versus SSC for SYTO 16-stained 6th-8th fractions of SEC and their mixture before (a) and after (b) DNase I digestion.

Note: The qEV single column (Izon, SP2101516) was equilibrated with 3.5 mL PBS before using. Then 150 µL EV isolate was pipetted onto the column, and fractions were immediately collected with a volume of 200 µL into each tube. PBS was used to elute EVs during the purification process. Based on the manufacturer's instructions, the first five fractions were discarded and the 6th–8th fractions were collected. The 6th-8th fraction and their equal volume mixture were analyzed on the nFCM before and after DNase I treatment. We can see from the results shown below although there exists some variation in the population ratios of cell-free DNA, debris or DNA$^-$ EVs, and DNA$^+$ EVs, none of the 6th - 8th fraction can provide EV preparation that does not have cell-free DNA.

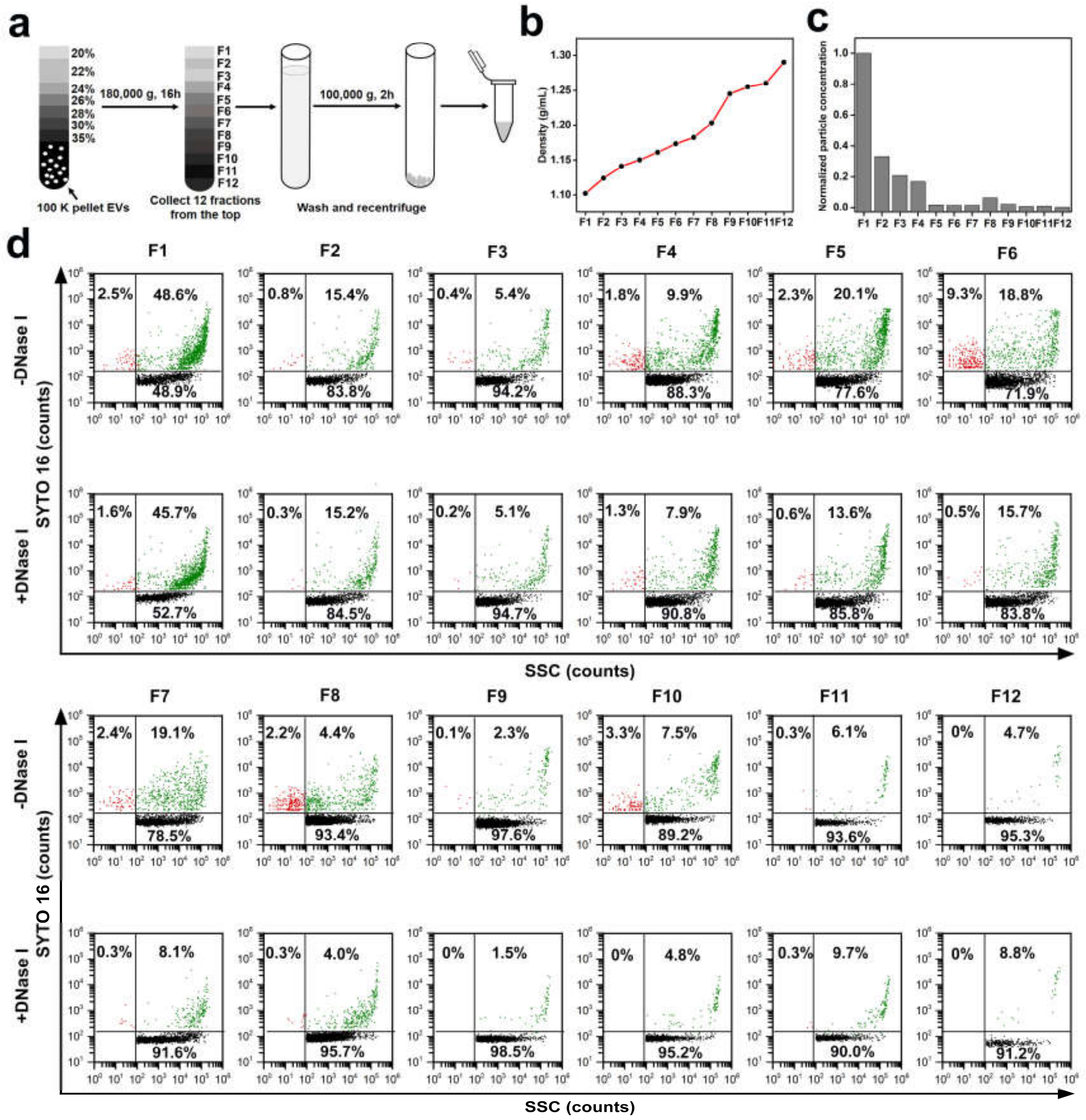

Figure S14. nFCM examination of the effectiveness of density gradient ultracentrifugation in separating cell-free DNA from EVs for the EV preparation obtained from the CCCM of HCT-15 cells by ultracentrifugation. (a) The schematic flow chart for density gradient ultracentrifugation. (b) The measured density for each iodixanol fraction. (c) Normalized particle concentration of each fraction. (d) Bivariate dot-plots of SYTO16 fluorescence versus SSC for EVs isolated from different iodixanol fractions before and after DNase I treatment.

Note: Freshly prepared CCCM from HCT-15 cells (60 mL) was divided equally into five tubes and centrifuged at 100,000 × g for 2 h at 4°C (Optima XE-90 ultracentrifuge with a SW 41Ti rotor, Beckman Coulter). All EV pellets were combined into a centrifuge

tube and suspended in 12 mL of PBS, followed by a second ultracentrifugation at 100,000 × g for 2 h at 4°C. Afterwards, the supernatant was discarded, and the EVs were resuspended in 1 mL of PBS. A total of 1 mL EV sample was mixed with 3 mL of 60% iodixanol (OptiPrepTM, 00119) and laid at the bottom of the centrifuge tube, and 1 mL layers of 35%, 30%, 28%, 26%, 24%, 22%, 22%, and 20% iodixanol were subsequently overlaid forming a discontinuous gradient. The sample was ultracentrifuged at 180,000 × g (Optima XE-90 ultracentrifuge with a SW 41Ti rotor, Beckman Coulter) for 16 h. Fractions of 1 mL were collected from the top to the bottom, and the density of each iodixanol fraction was measured by weighting. Next, the sample of different fractions was transferred to a new tube, diluted, washed with PBS (up to 12 mL), and ultracentrifuged at 100,000 × g for 2 h (Optima XE-90 ultracentrifuge with a SW 41Ti rotor, Beckman Coulter). Afterwards, the supernatant was discarded, and the EVs were resuspended in 200 μL of PBS. These samples were stained with SYTO 16 and analyzed on the nFCM before and after DNase I treatment. The particle concentration of each fraction was also measured. We can see that although density gradient ultracentrifugation can efficiently separate cell-free DNA from EVs, the EV-DNA adhered to the outer membrane of EVs was also detached from the surface owing to the harsh separation process.

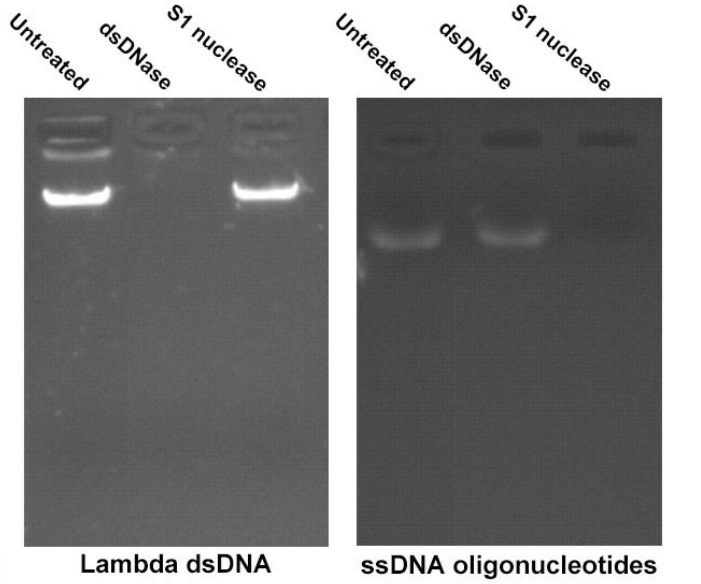

Figure S15. The functionality and specificity of dsDNase and S1 nuclease were verified by using purified Lambda dsDNA and ssDNA oligonucleotides as the substrates.

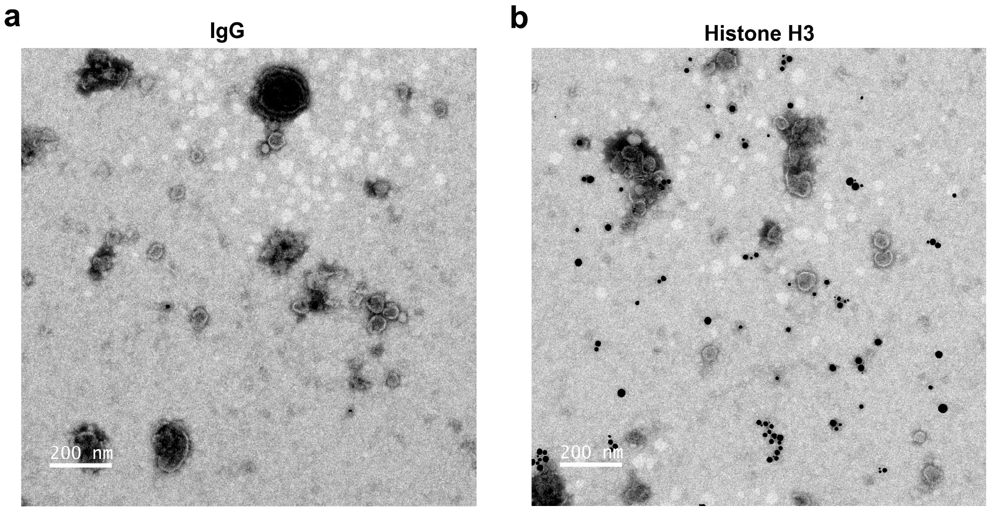

Figure S16. Immuno-gold labelling of histone H3 in EV isolates. (a) EVs were incubated with rabbit IgG and a 10 nm gold-conjugated goat anti-rabbit IgG (H+L). (b) EVs were incubated with rabbit anti-histone H3 antibody and a 10 nm gold-conjugated goat anti-rabbit IgG (H+L).

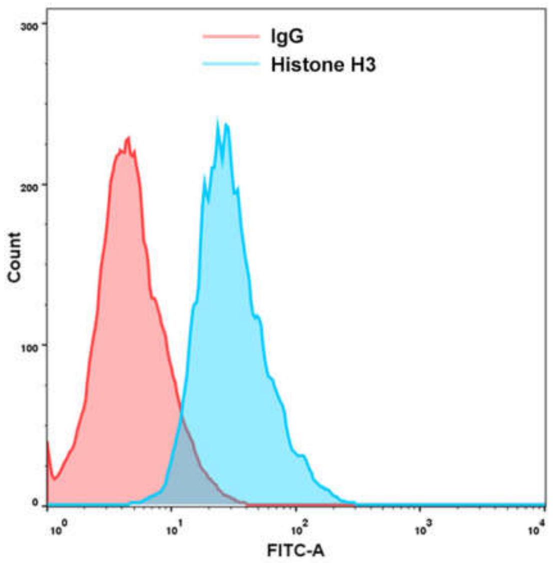

Figure S17. HCT-15 cells were stained with Alexa Fluor 488 conjugated monoclonal antibodies (MAbs) specific to histone H3 and detected by BD FACSAria IIIu Cytometer.

Note: About 1 × 10^6 HCT-15 cells were collected and centrifuged at 800 × g for 5 min at 4°C. Cells were resuspended in 100 µL 4% PFA and incubated at room temperature for 30 min. The PFA was washed away with 1 mL PBS by ultracentrifugation at 800 g for 5 min at 4 °C. Subsequently, cells were resuspended in 100 µL of 0.2% Tween 20, incubated for 15 min at room temperature. Cells were centrifuged at 800 × g for 5 min at 4°C, and resuspended in 100 µL PBS. Then, 5 µg/mL of AF488-conjugated mouse anti-human histone H3 antibody (Thermo Fisher, MA531759) was added. The mixture was incubated at 37°C for 30 min and then washed twice with 1 mL PBS by ultracentrifugation at 800 × g for 5 min at 4°C. The pellet was resuspended in 500 µL PBS for BD FACSAria IIIu Cytometer analysis.

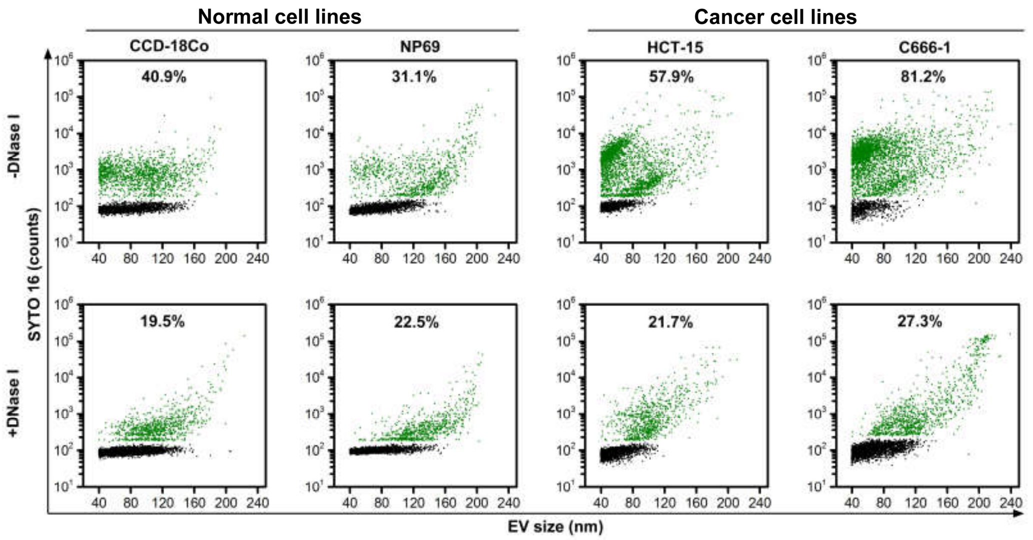

Figure S18. nFCM analysis of DNA on EVs isolated from normal cell lines (CCD-18Co and NP69) and cancer cell lines (HCT-15 and C666-1) without (a) or with (b) DNase I treatment prior to SYTO 16 staining.
