# Supplementary material for: Analysis of extracellular vesicle DNA at the single‐vesicle level by nano‐flow cytometry
Source: J Extracell Vesicles. 2022 Apr 4;11(4):e12206. doi: 10.1002/jev2.12206 (PMC8977970; doi:10.1002/jev2.12206)
Supplement: Supplementary file 3 — Supporting information. [file JEV2-11-e12206-s001.doc]

| **Requirement** | **Please Include Requested Information** |
| --- | --- |
| 1.1. Purpose | EV-DNA analysis at the single-vesicle level to reveal the large intrinsic heterogeneity among individual EVs and overcome the ambiguity in interpreting data obtained from ensemble-averaged measurements. Specifically, to measure the percentage of DNA^+^ EVs along with the DNA content distribution versus EV size, the differentiation between ssDNA and dsDNA, the relevance of EV-DNA to histone proteins, and the alteration of DNA content under anticancer drug treatment. |
| 1.2. Keywords | Extracellular vesicles; exosomes; microvesicles; single particles; DNA; nuclease; histones; nano-flow cytometry |
| 1.3. Experiment variables | The DNA content of EVs with or without DNase I treatment were analyzed by nFCM. Scatter-based triggering was used for the detection of EVs. |
| 1.4. Organization name and address | Department of Chemical Biology, MOE Key Laboratory of Spectrochemical Analysis & Instrumentation, Key Laboratory for Chemical Biology of Fujian Province, Collaborative Innovation Center of Chemistry for Energy Materials, Department of Chemical Biology, College of Chemistry and Chemical Engineering, Xiamen University, Xiamen 361005, People's Republic of China |
| 1.5. Primary contact name and email address | Prof. Dr. Xiaomei Yan, xmyan@xmu.edu.cn |
| 1.6. Date or time period of experiment | 2018.03 – 2021.10 |
| 1.7. Conclusions | Through simultaneous side-scatter and fluorescence detection of single particles and with the combination of enzymatic treatment, this study revealed that: 1) naked DNA or DNA associated with non-vesicular entities is abundantly presented in EV samples prepared from cell culture medium by ultracentrifugation; 2) the quantity of EV-DNA in individual EVs exhibits large heterogeneity and the population of DNA positive (DNA+) EVs varies from 30% to 80% depending on the cell type; 3) external EV-DNA is mainly localized on relatively small size EVs (e.g., < 100 nm for HCT-15 cell line) and the secretion of external DNA+ EVs can be significantly reduced by exosome secretion pathway inhibition; 4) internal EV-DNA is mainly packaged inside the lumen of relatively large EVs (e.g., 80–200 nm for HCT-15 cell line); 5) double-stranded DNA is the predominant form of both the external and internal EV-DNA; 6) histones (H3) are not found in EVs and EV-DNA is not associated with histone proteins; and 7) genotoxic drug induces an enhanced release of DNA containing EVs, and the number of both external DNA+ EVs and internal DNA+ EVs as well as the DNA content in single EVs increase markedly. This study provides direct and conclusive experimental evidence for an in-depth understanding of how DNA is associated with EVs. |
| 1.8. Quality control measures | We analyzed EV isolates stained with SYTO 16 by nFCM at the single-vesicle level according to the MIFlowCyt-EV guideline. Assay controls (buffer only, buffer with reagent, and unstained EV samples) were analyzed. Monodispersed silica nanoparticles of known size were analyzed. A mixture containing 400, 2000, and 5000-bp of DNA fragments was stained with SYTO 16 and analyzed. |
| 2.1.1.1. (2.1.2.1., 2.1.3.1.) Sample description | Extracellular vesicles isolated from several human cell lines (HCT-15, CCD-18Co, NP69 and C666-1) by ultracentrifugation |
| 2.1.1.2. Biological sample source description | The human colorectal cancer cell line (HCT-15) and a normal human colon fibroblast cell line (CCD-18Co) were purchased from the American Type Culture Collection (ATCC). The human nasopharyngeal epithelial cell line (NP69) and nasopharyngeal carcinoma cell line (C666-1) were generously provided by Professor George Tsao (University of Hong Kong, China). |
| 2.1.1.3. Biological sample source organism description | Human |
| 2.1.2.2. Environmental sample location | N/A |
| 2.3. Sample treatment description | The conditioned cell culture medium (CCCM) was succumbed to differential ultracentrifugation. EVs were subjected to nuclease digestion or Triton X-100 treatment. |
| 2.4. Fluorescence reagent(s) description | | Analyte | Reagent | Fluorochrome | Manufacturer | Catalog number | | --- | --- | --- | --- | --- | | DNA | SYTO 16 |  | ThermoFisher | S7578 | | CD9 | Anti-CD9 | PE | BD Biosciences | 555372 | | CD63 | Anti-CD63 | PE | BD Biosciences | 556020 | | CD81 | Anti-CD81 | PE | BD Biosciences | 555676 | | Actin | Anti-actin | AF488 | Proteintech | 60008-1-1g | | Histone H3 | Anti-Histone H3 | AF488 | Thermo Fisher | MA531759 | |
| 3.1. Instrument manufacturer | A laboratory-built nano-flow cytometer was used in present study. Note that this instrument is now commercially available from NanoFCM Inc. (Xiamen, China), and the Flow NanoAnalyzer shares same principle and comparable performance with the laboratory-built instrument. |
| 3.2. Instrument model | A laboratory-built nano-flow cytometer was used in present study. |
| 3.3. Instrument configuration and settings | 3.3.1 Light source: 20 mW 488-nm laser (OBIS 488LS, Coherent)  3.3.2 Optics filters and detectors: Two single-photon counting avalanche photodiodes (APDs) were used for the simultaneous detection of side scatter (bandpass filter: FF01-488/6) and green fluorescence (bandpass filter: FF01-525/45) of individual particles/EVs, respectively. |
| 4.1. List-mode data files | FC files and the analysis workspace have been uploaded FlowRepository and can be obtained at http://flowrepository.org/id/RvFrqAJW1Zmsju10WOSMpPd5I9LHSt152WtSuAr7q9PbomVF4GZhRBFqckljhkeU |
| 4.2. Compensation description | No compensation was applied. |
| 4.3. Data transformation details | The output signals from the APD detectors were counted using a National Instruments DAQ card (PCIe-6321). A custom program written with LabVIEW 2012 software was used for data acquisition and processing, and the bin width was set to 100 μs. The threshold levels for both the peak height (a digital discriminator level set to 3 times the standard deviation of the background) and the peak width of 0.2 ms and 0.3 ms was set as the criteria for burst (or peak) identification of SSC and FL signal, respectively. For each burst that satisfied the criteria, the integrated number of photons (background subtracted) was stored as the burst area for the histogram or dot-plot construction. Unless stated otherwise, each distribution histogram or dot-plot was derived from the data collected over 1 min. |
| 4.4.1. Gate description | On the bivariate dot-plot of SYTO 16 FL versus SSC, a clear separation can be obtained for the three populations of cell-free DNA, debris or DNA- EVs, and DNA+ EVs. |
| 4.4.2. Gate statistics | Plots show the population ratio for each population on the described parameter. Three replicate experiments were conducted. See figures of the manuscript and results section for further details. |
| 4.4.3. Gate boundaries | Gates boundaries were set according to each population. |
